# Supplementary figures and images for: DICER1 hotspot mutation induces 3p microRNA gain of function via Argonaute strand switch
Source: Nat Struct Mol Biol. 2025 Nov 4;32(12):2542–52. doi: 10.1038/s41594-025-01671-w (PMC12700799; doi:10.1038/s41594-025-01671-w)

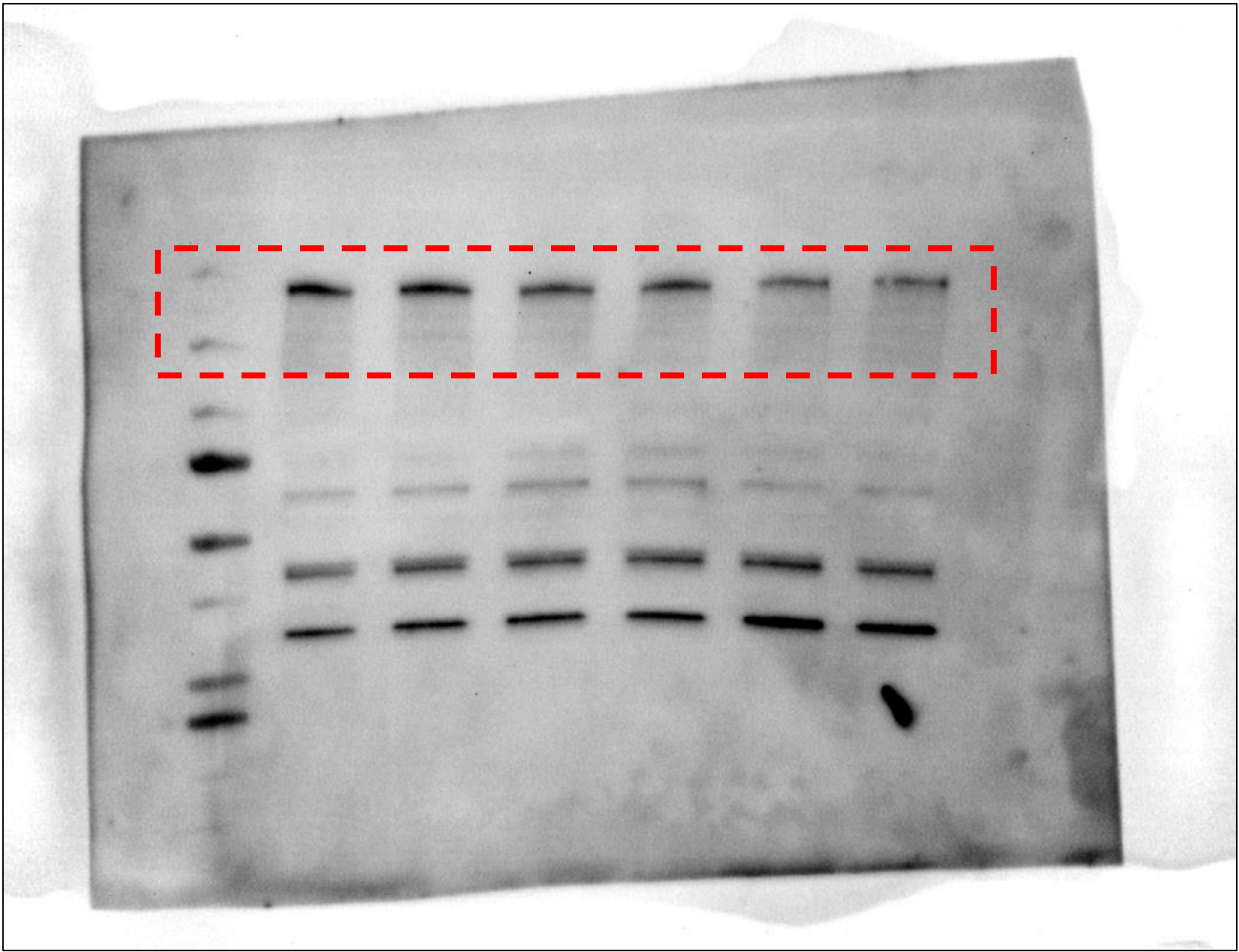

Dicer

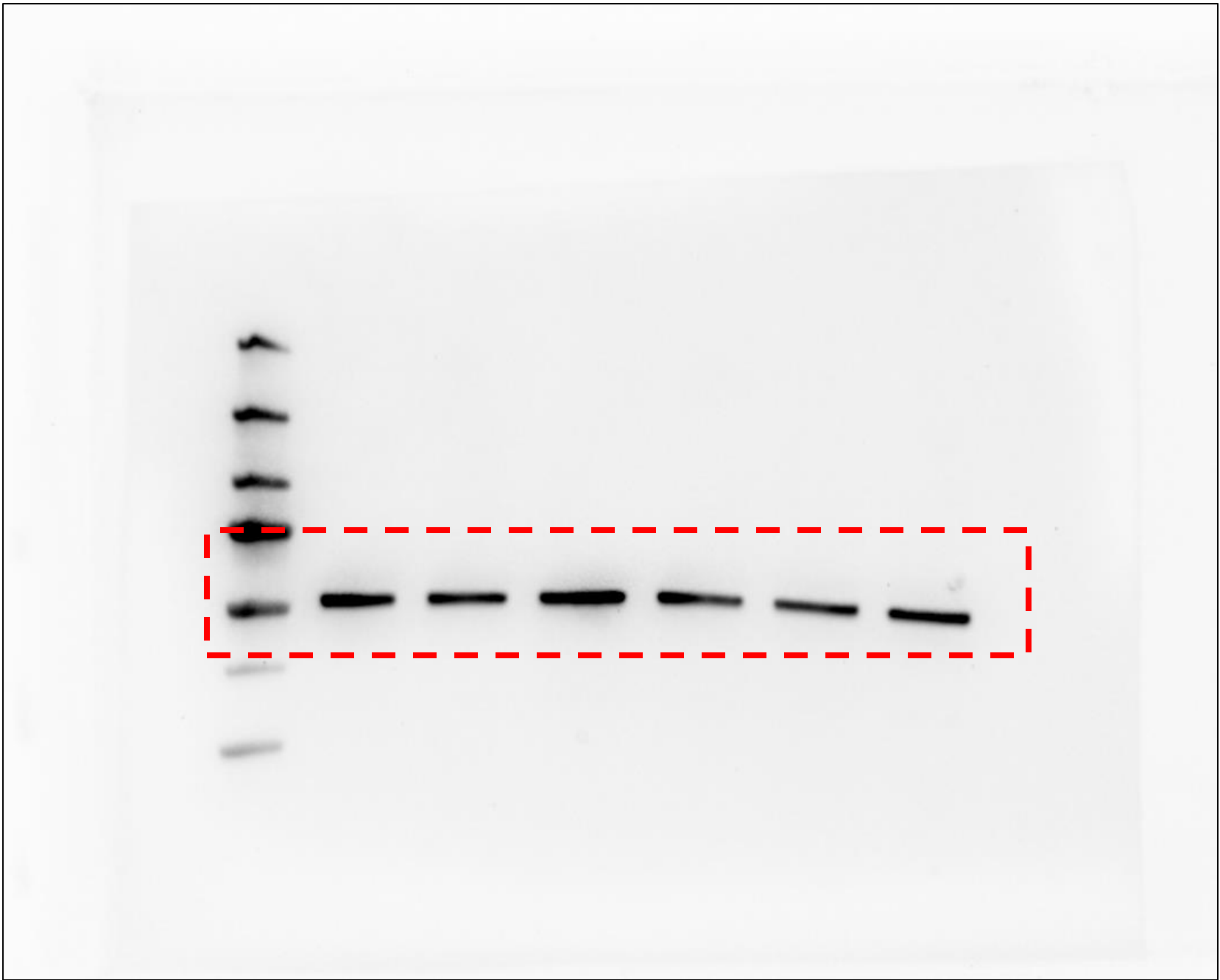

$\alpha$ -tubulin

Figure 1d

Supplement: Supplementary file 4 — Unprocessed western blot image data for Fig. 1. [file 41594_2025_1671_MOESM4_ESM.pdf]

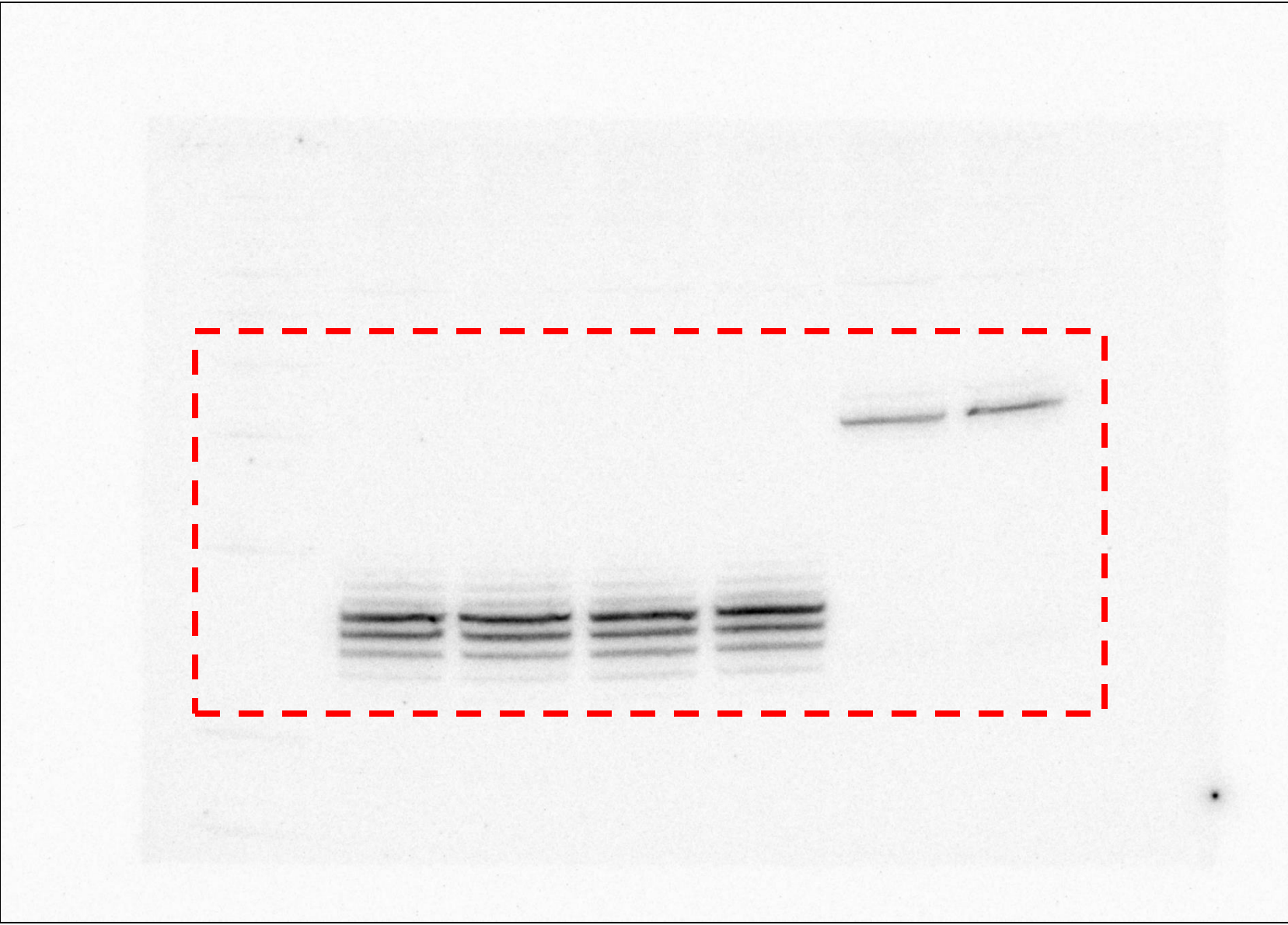

**miR-7a-5p**

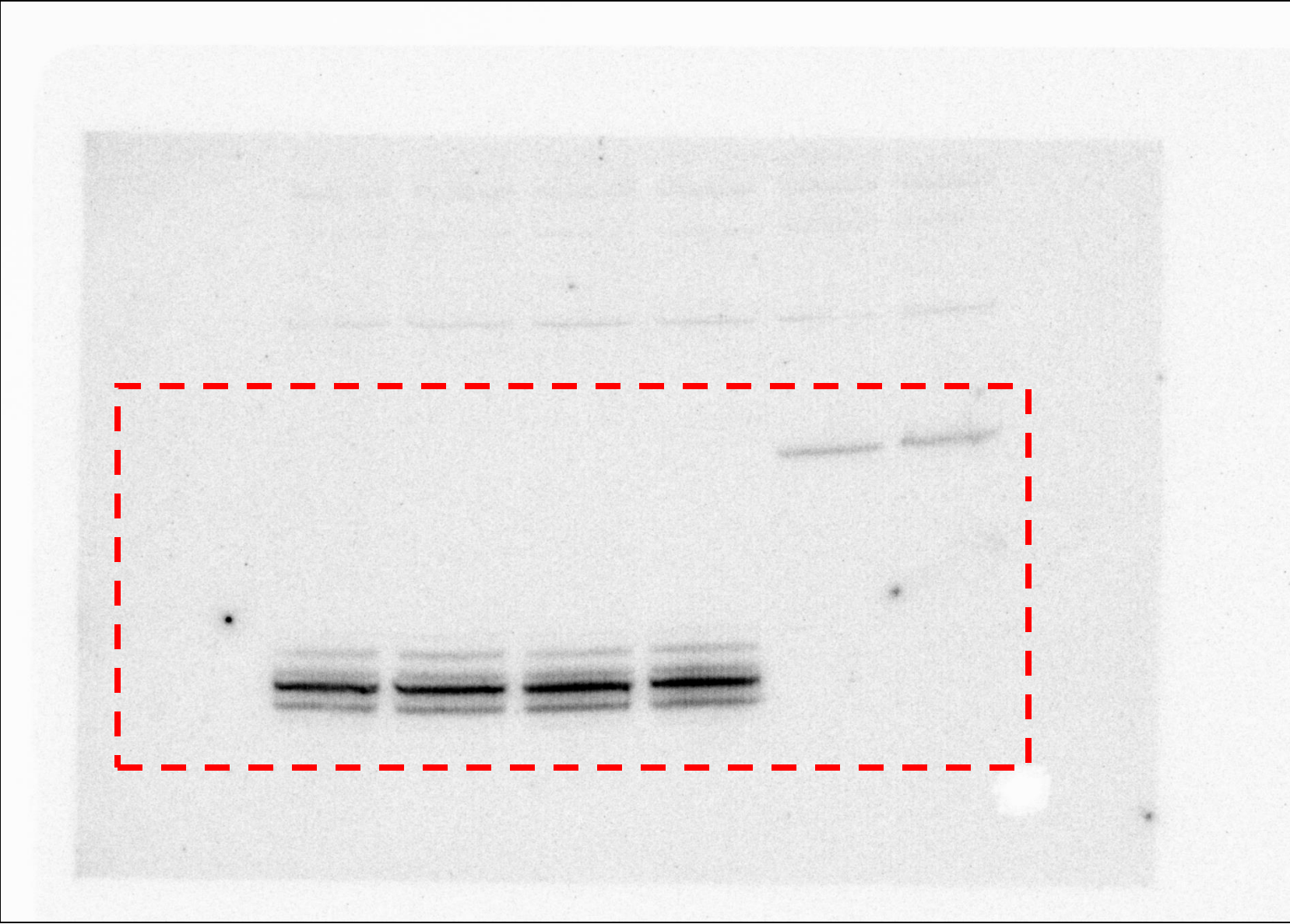

**miR-15b-5p**

**Figure 2d**

Section presented

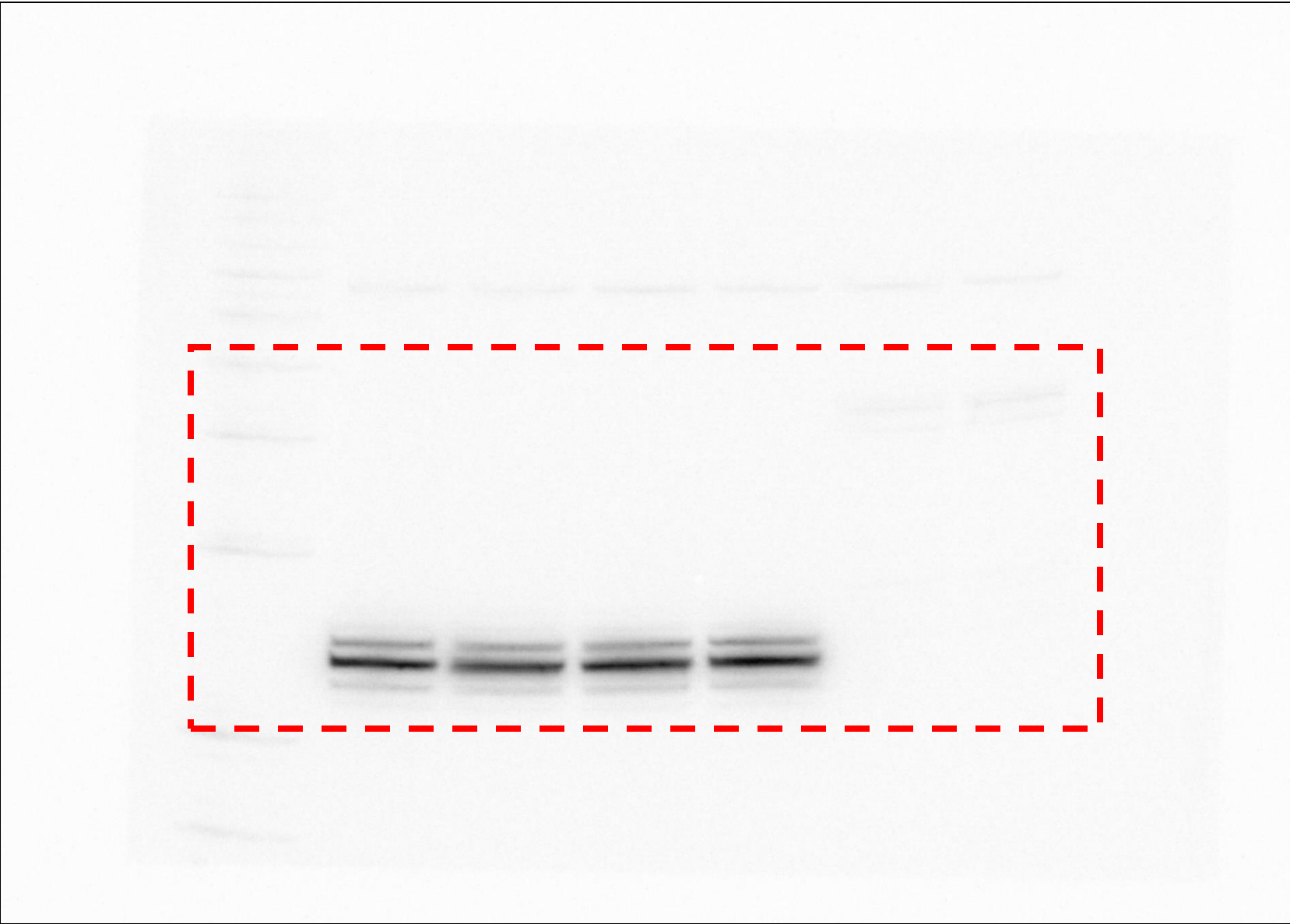

**miR-16-5p**

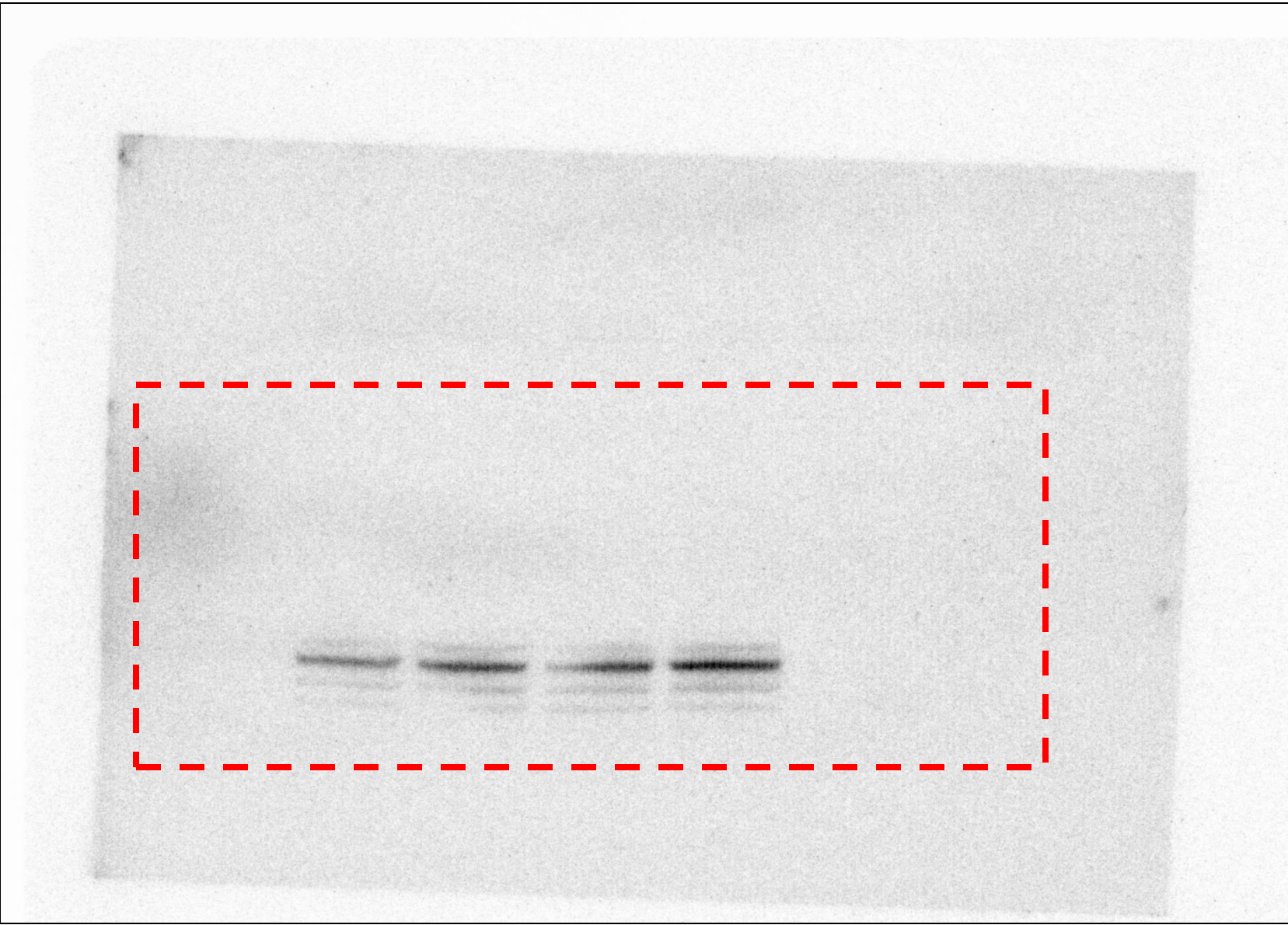

**miR-17-5p**

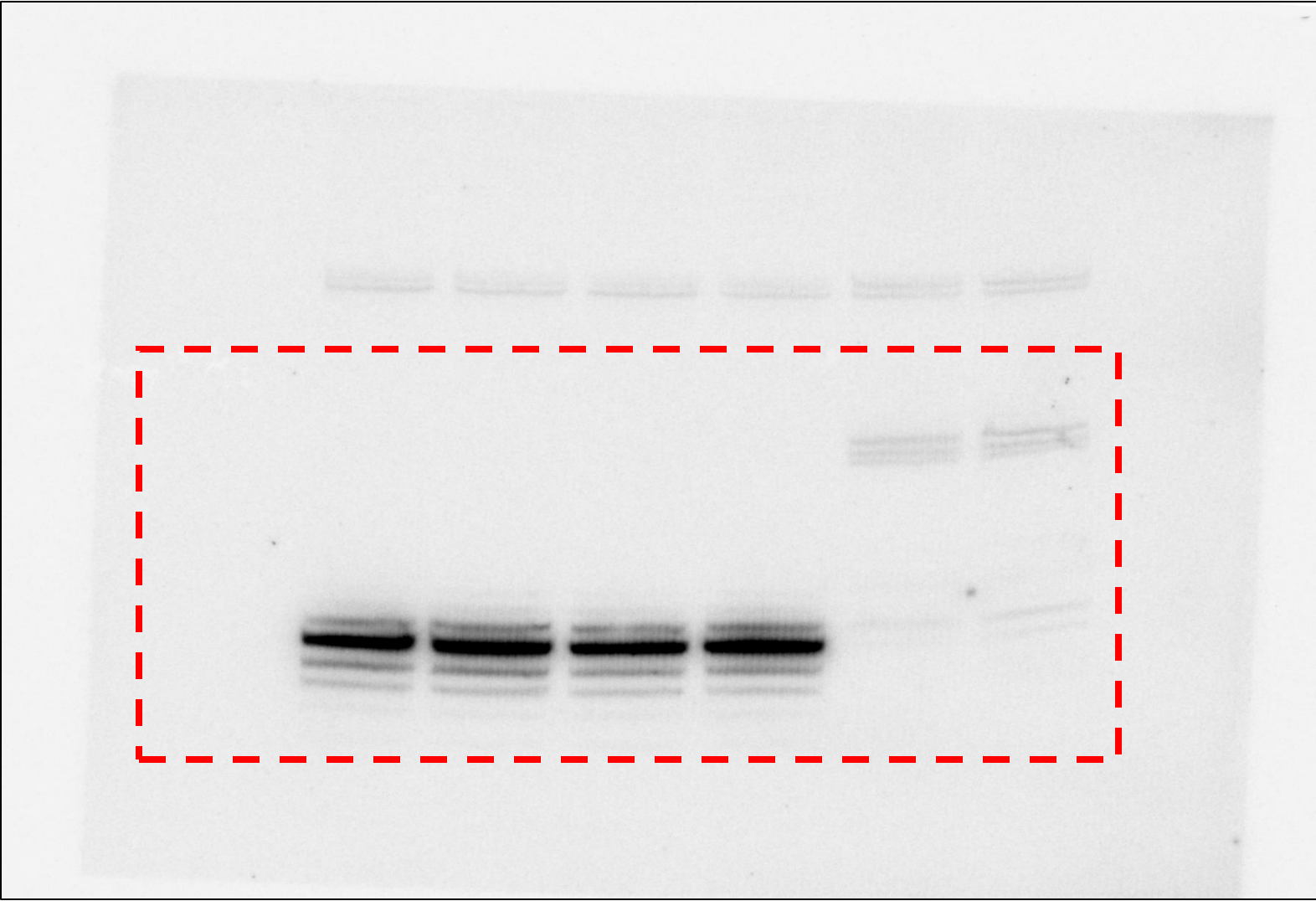

**miR-20b-5p**

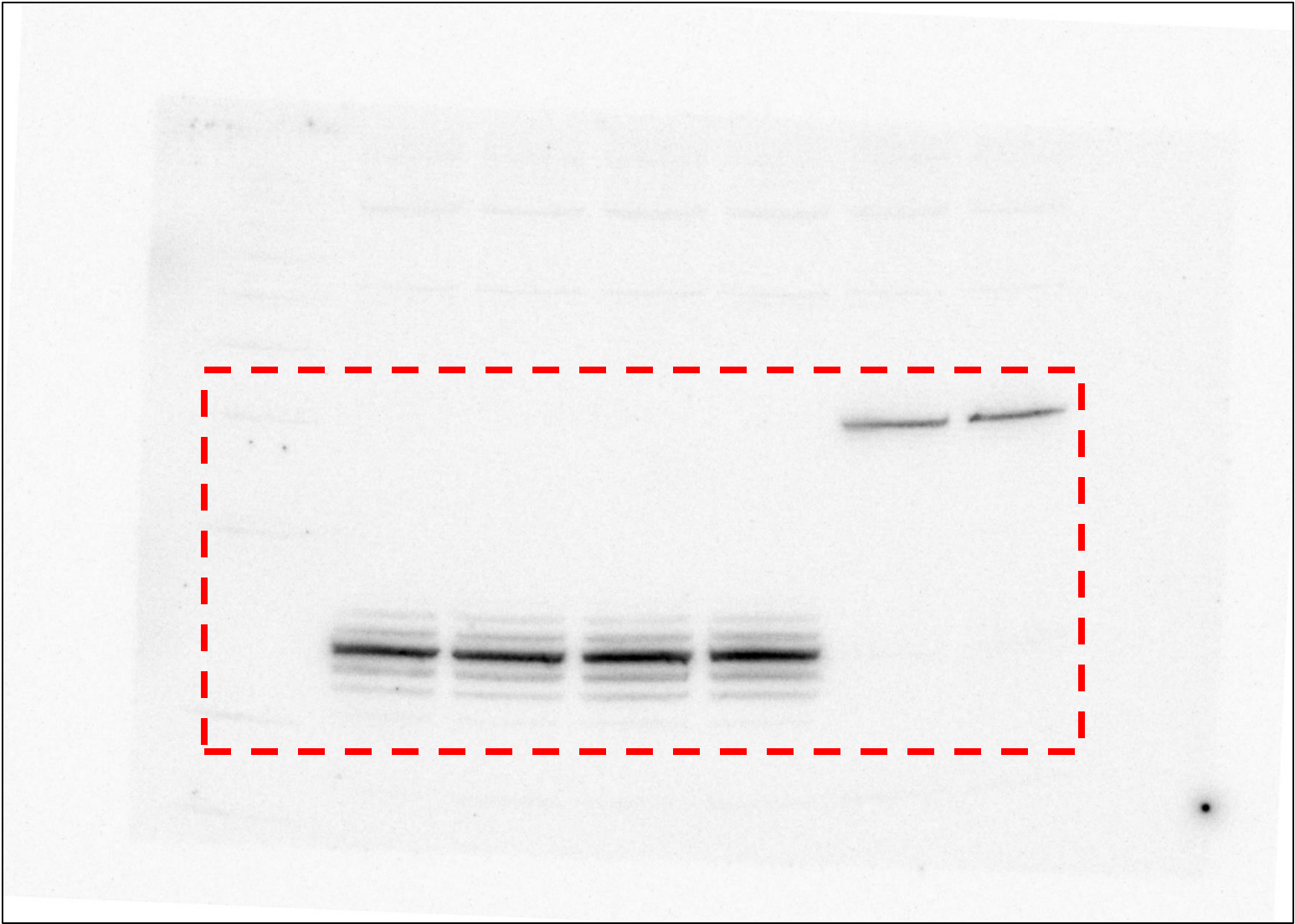

**miR-26a-5p**

**Figure 2d (continued)**

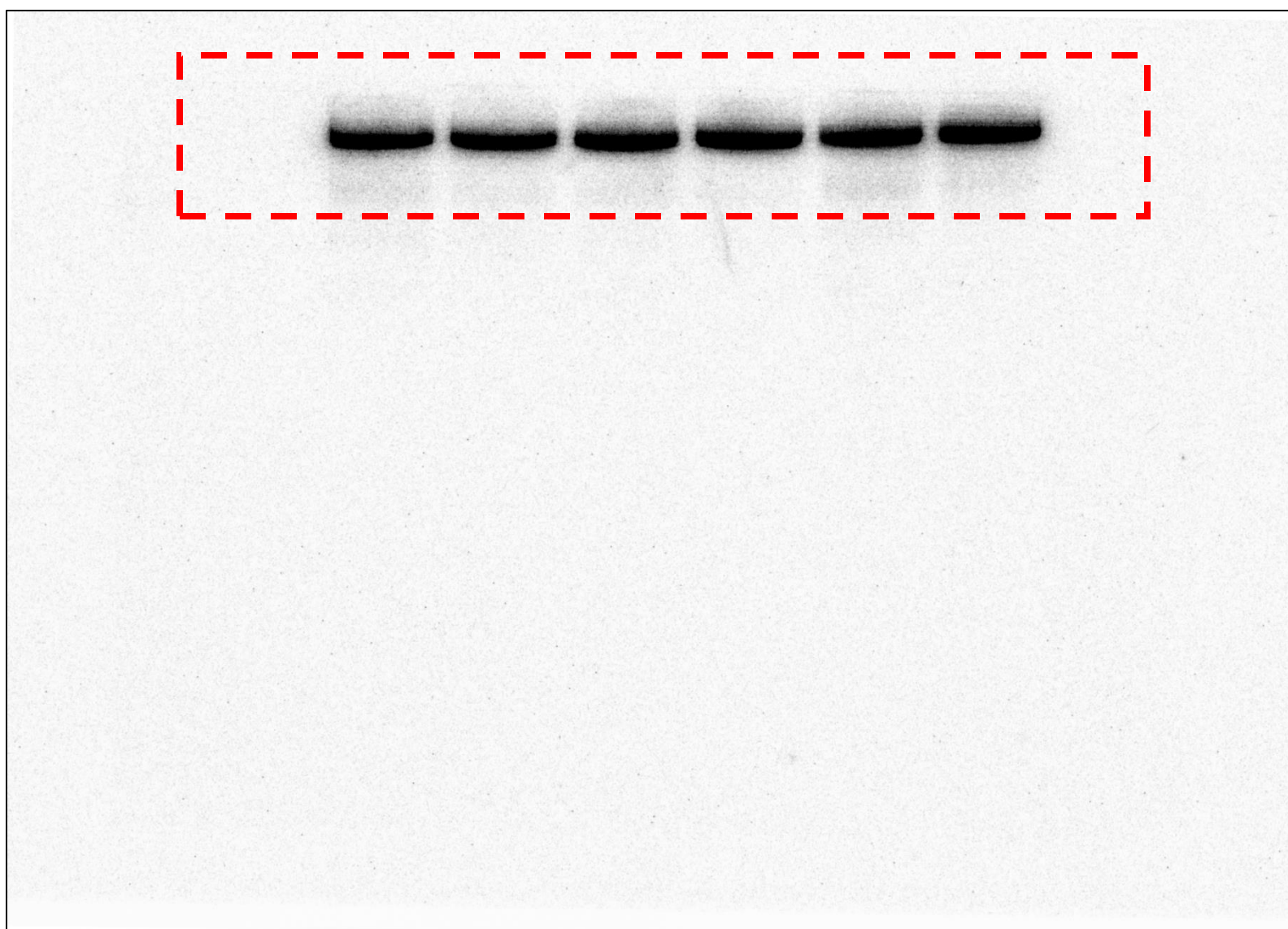

**U6**

Supplement: Supplementary file 6 — Unprocessed northern blot image data for Fig. 2. [file 41594_2025_1671_MOESM6_ESM.pdf]

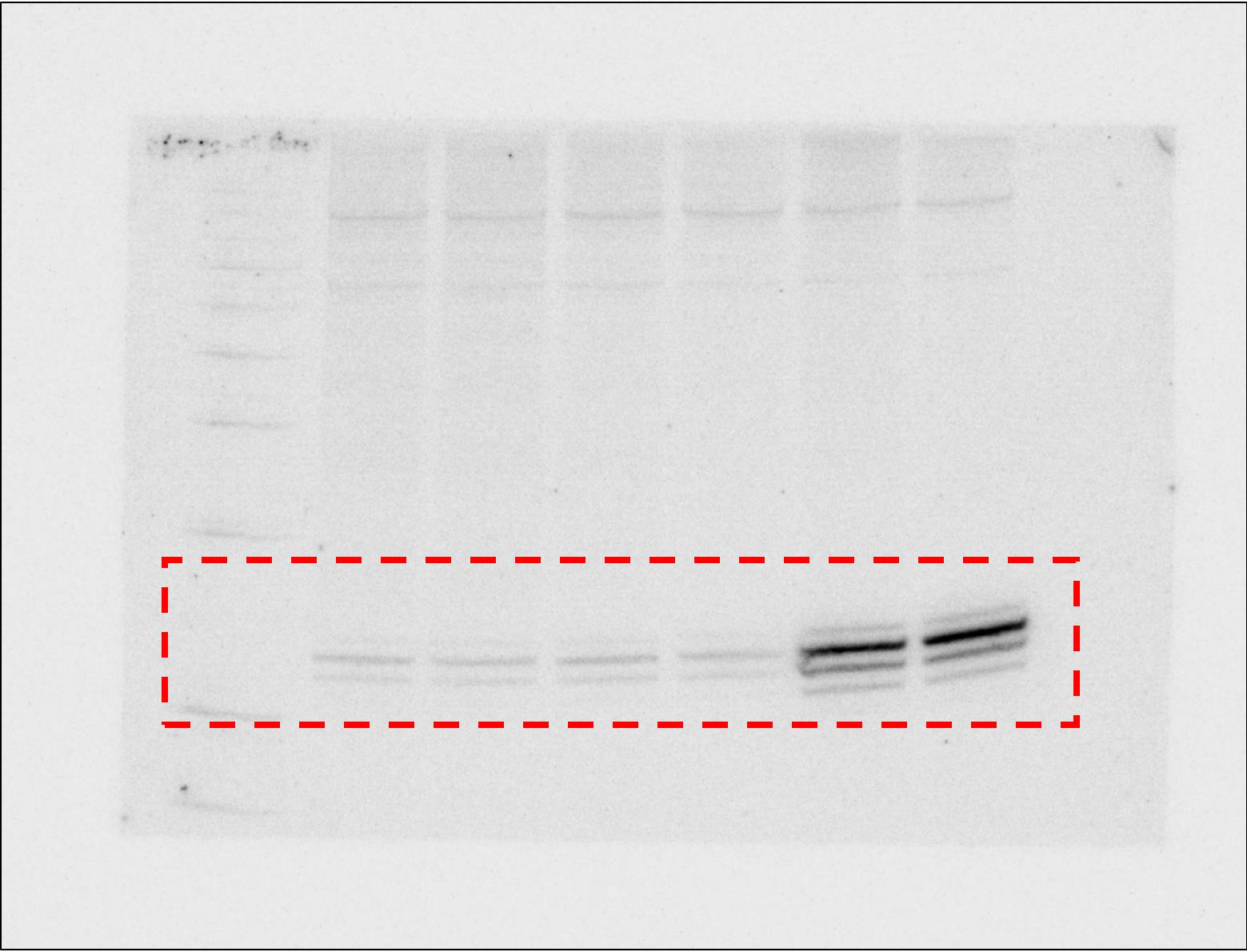

**miR-7a-1-3p**

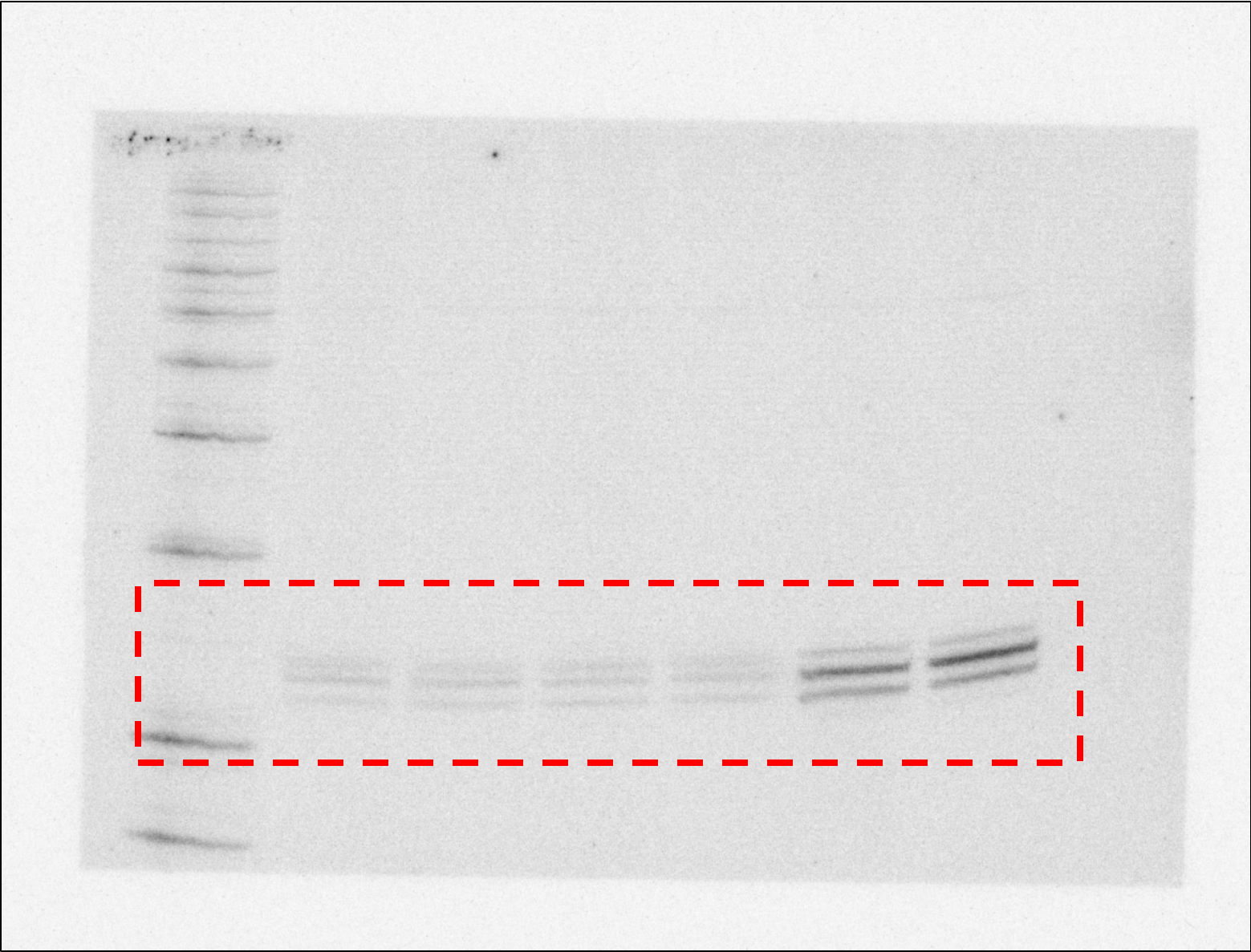

**miR-15b-3p**

**Figure 3d**

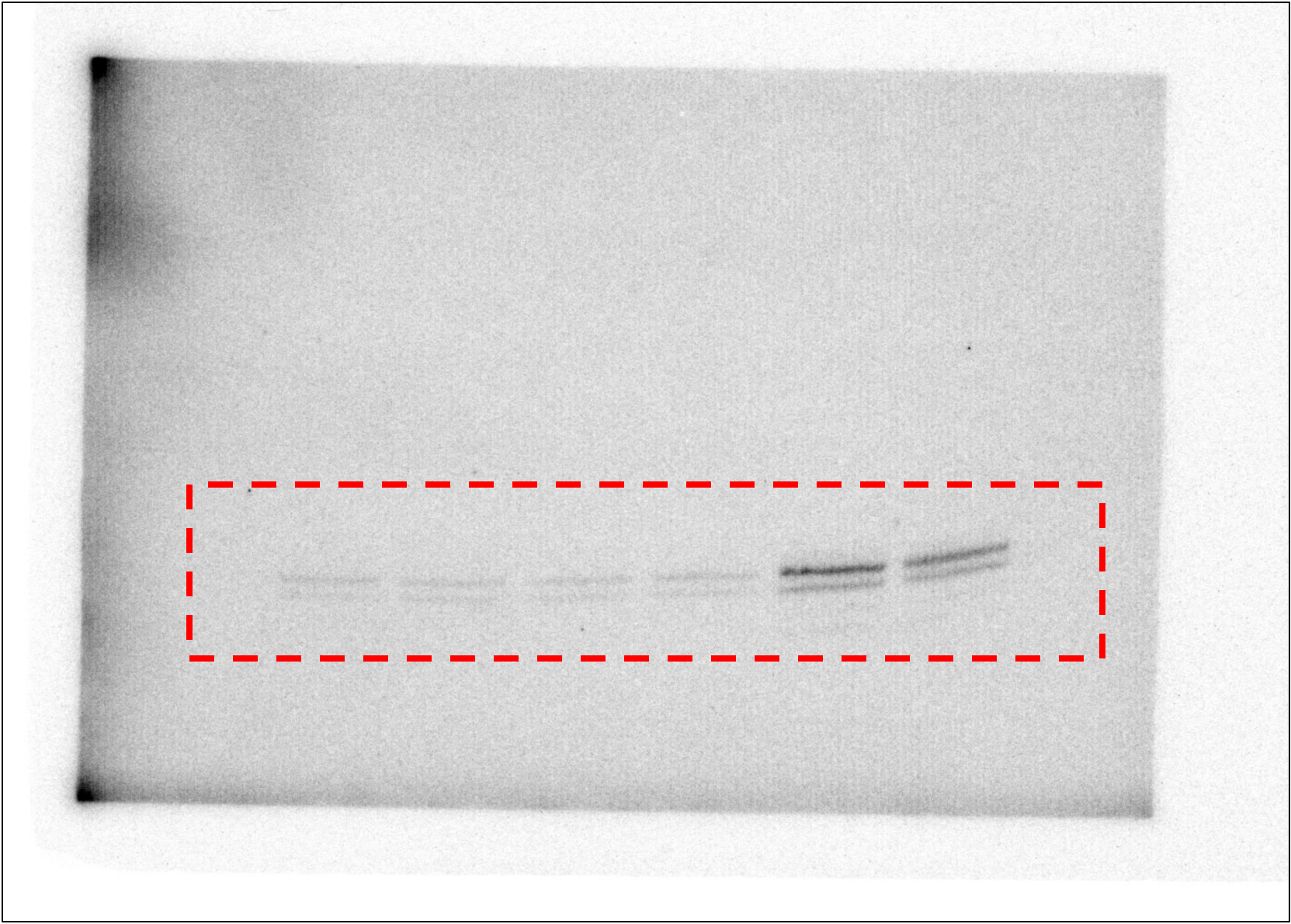

**miR-17-3p**

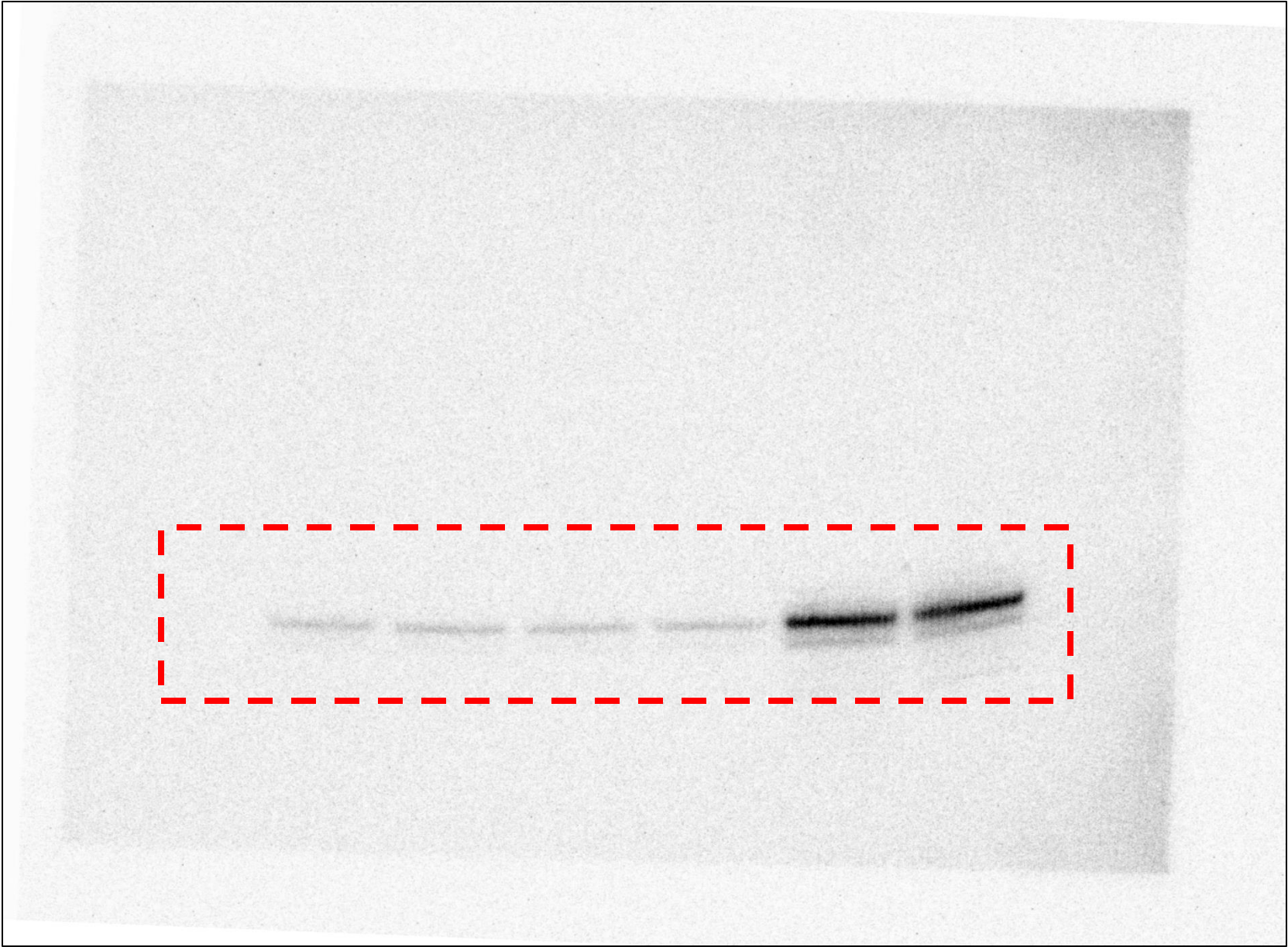

**miR-20b-3p**

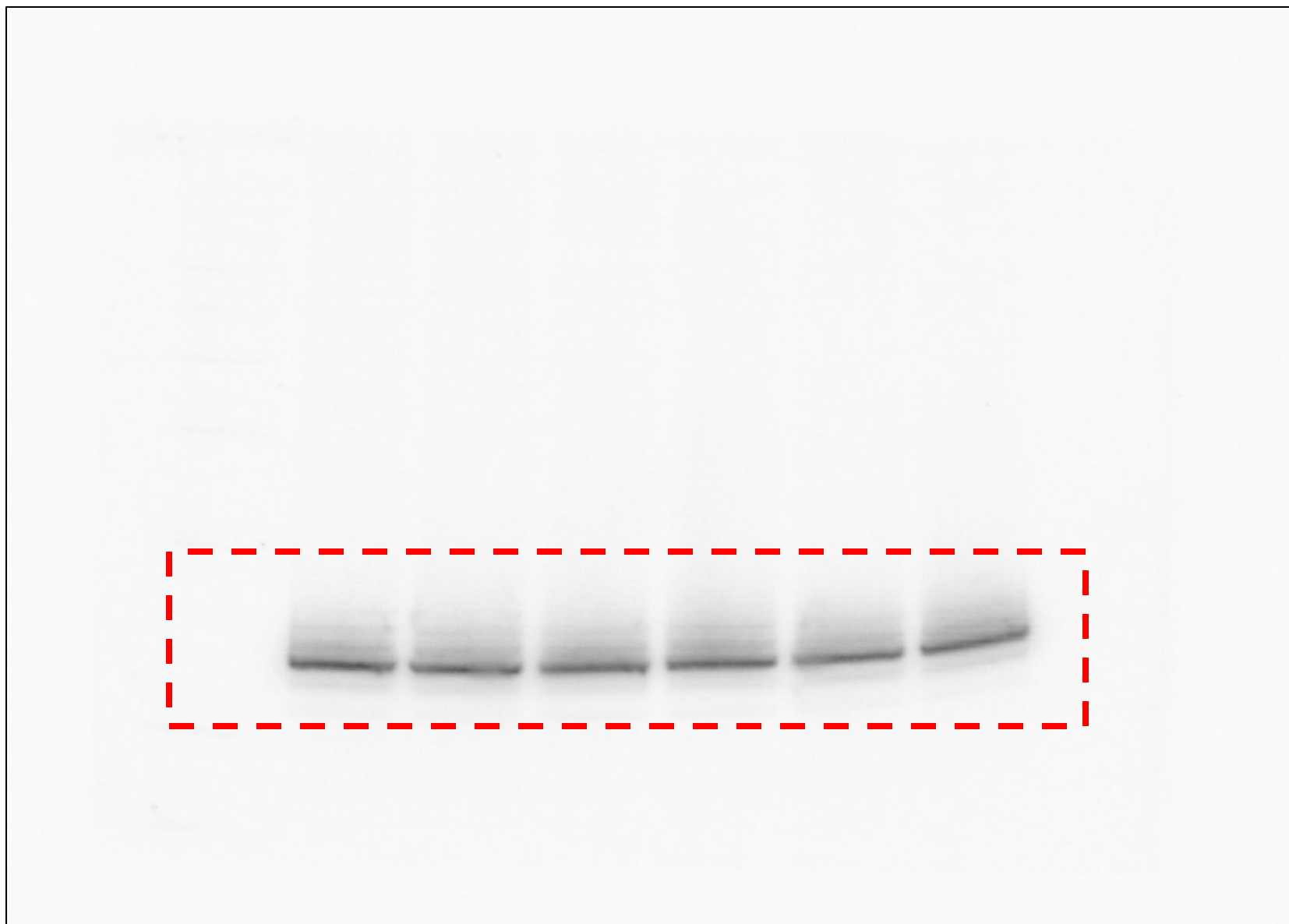

**miR-92a-3p**

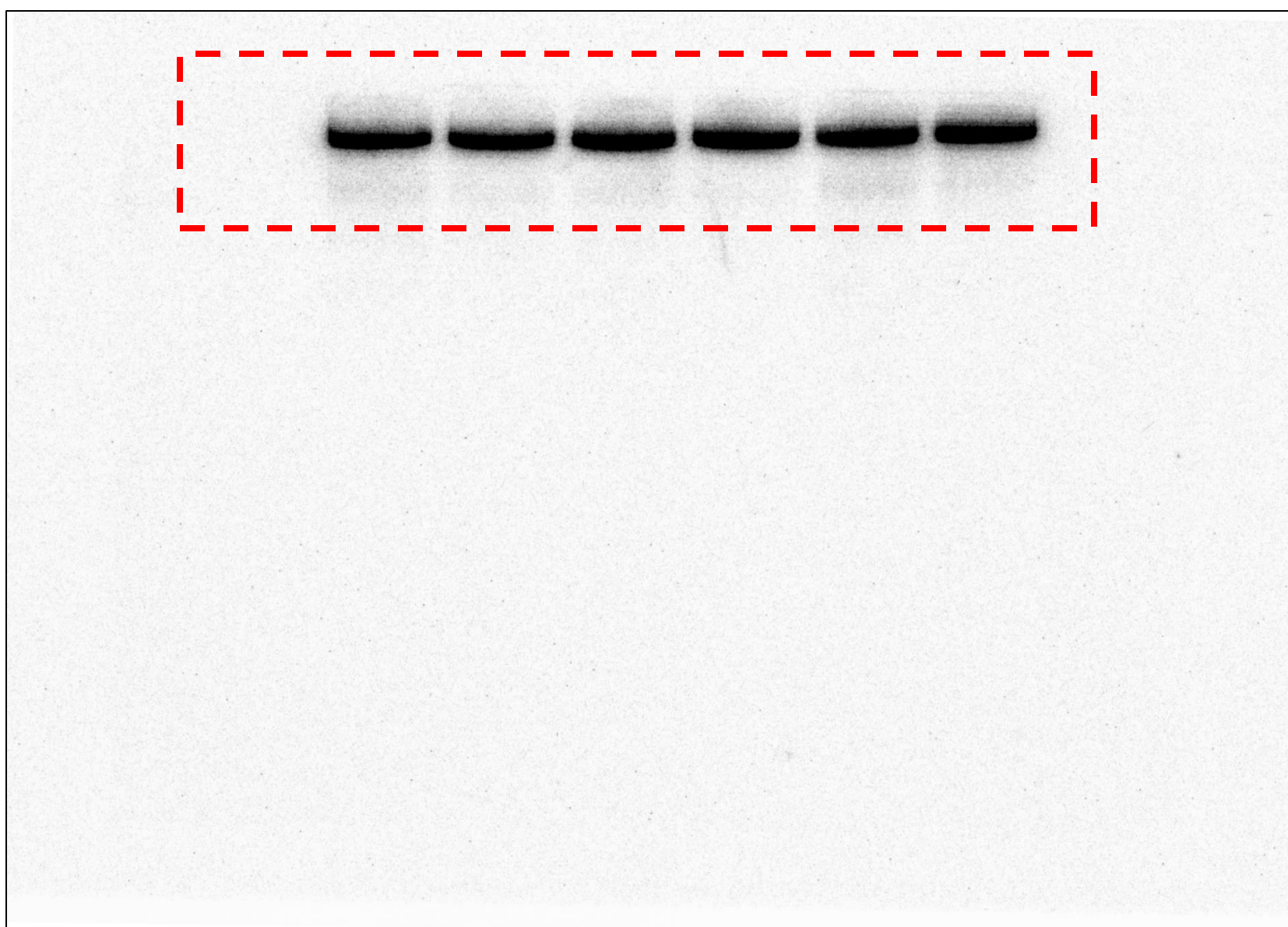

**U6**

**Figure 3d (continued)**

Supplement: Supplementary file 8 — Unprocessed northern blot image data for Fig. 3. [file 41594_2025_1671_MOESM8_ESM.pdf]

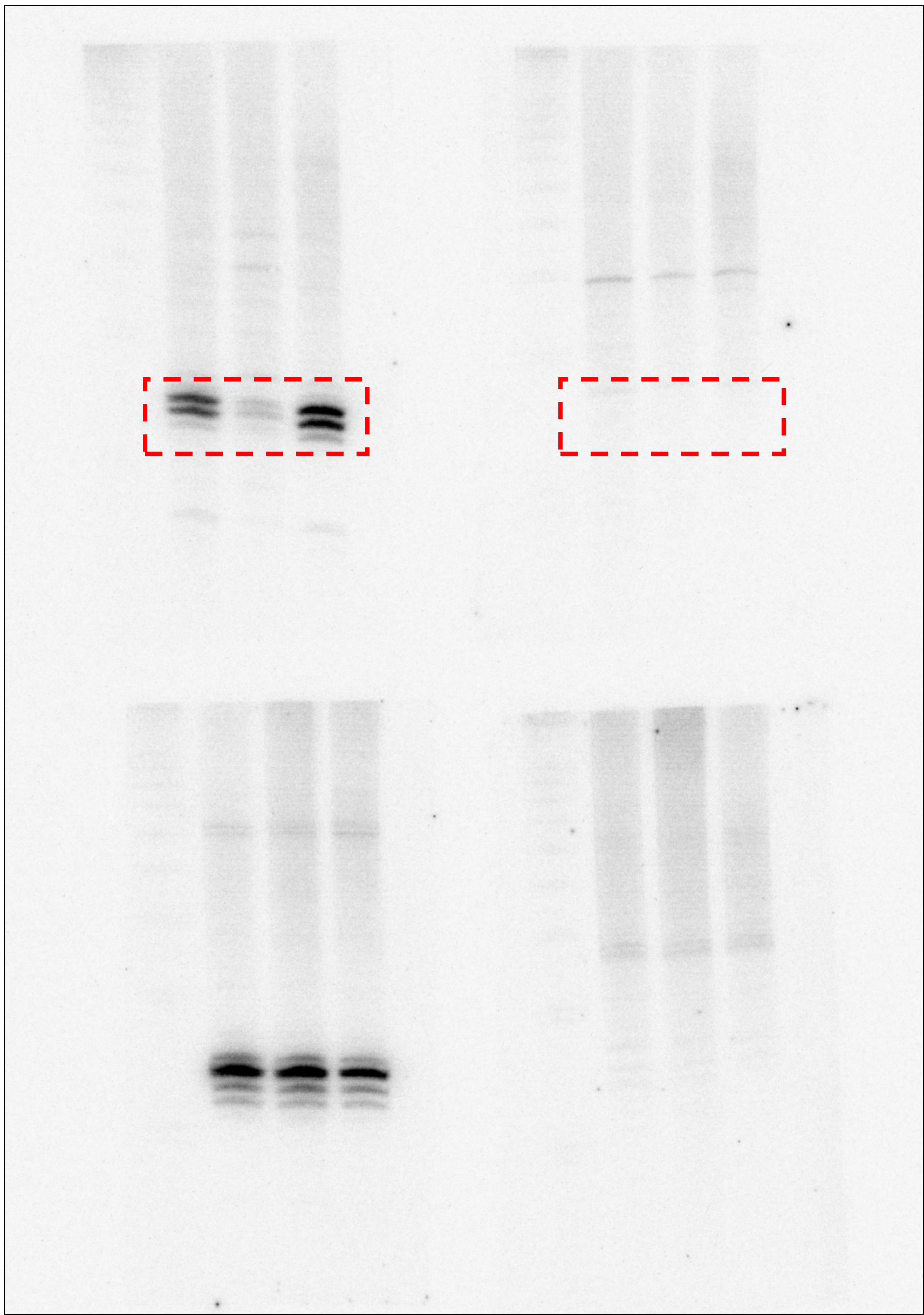

**Figure 5e (top panels)**

Section presented

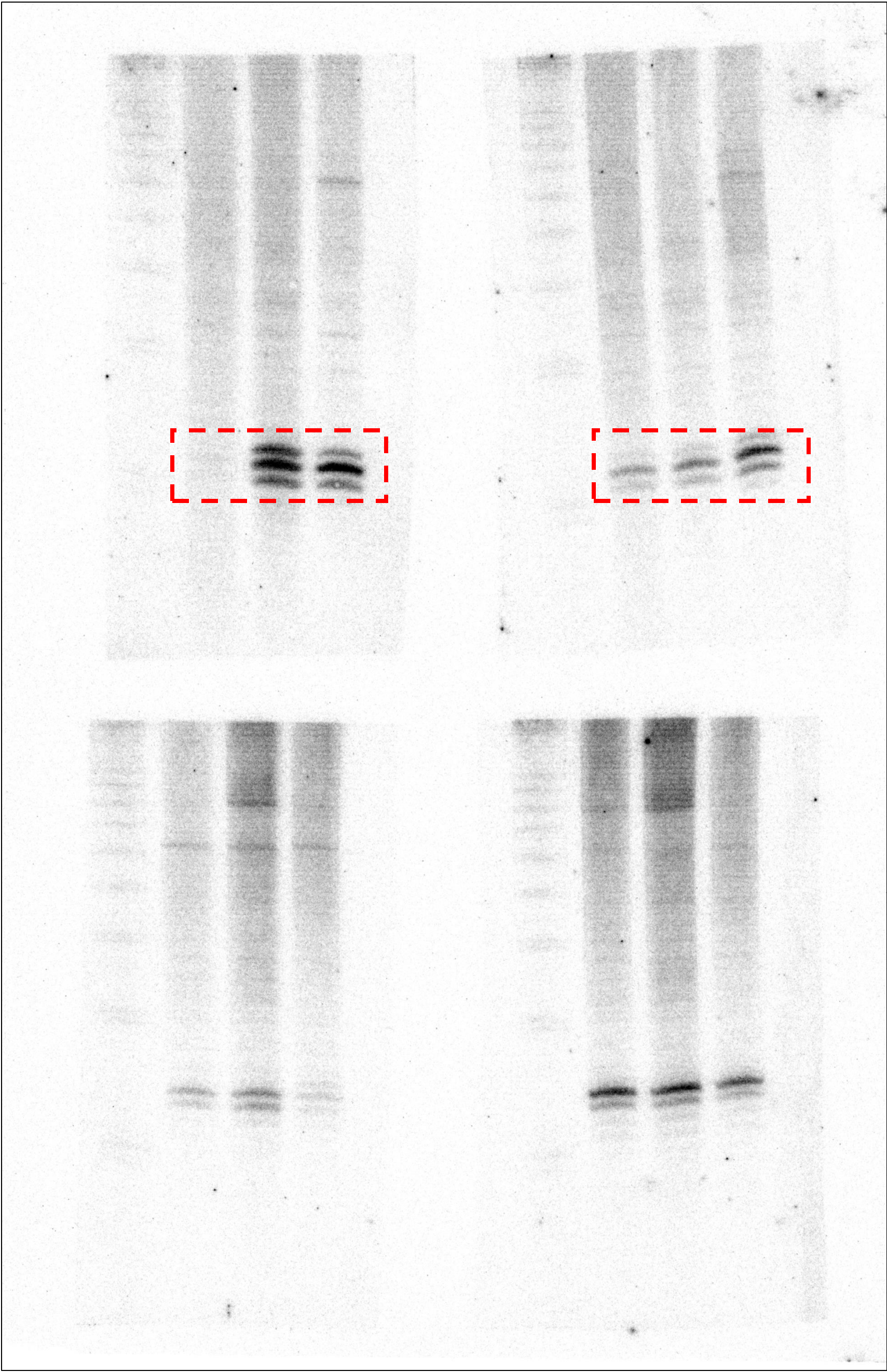

**Figure 5e (bottom panels)**

Section presented

Supplement: Supplementary file 12 — Unprocessed northern blot image data for Fig. 5. [file 41594_2025_1671_MOESM12_ESM.pdf]

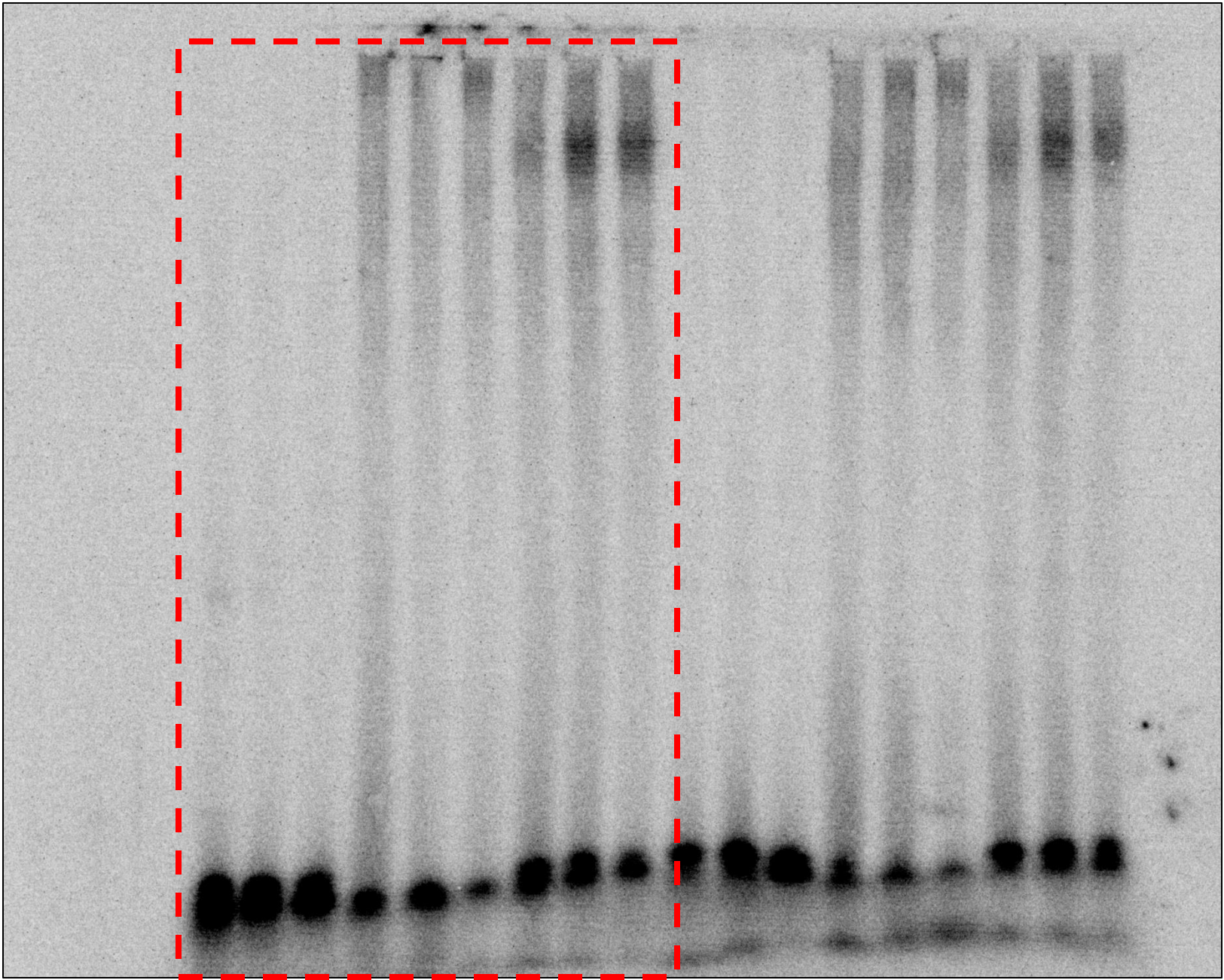

**Gel i**

**Gel iii**

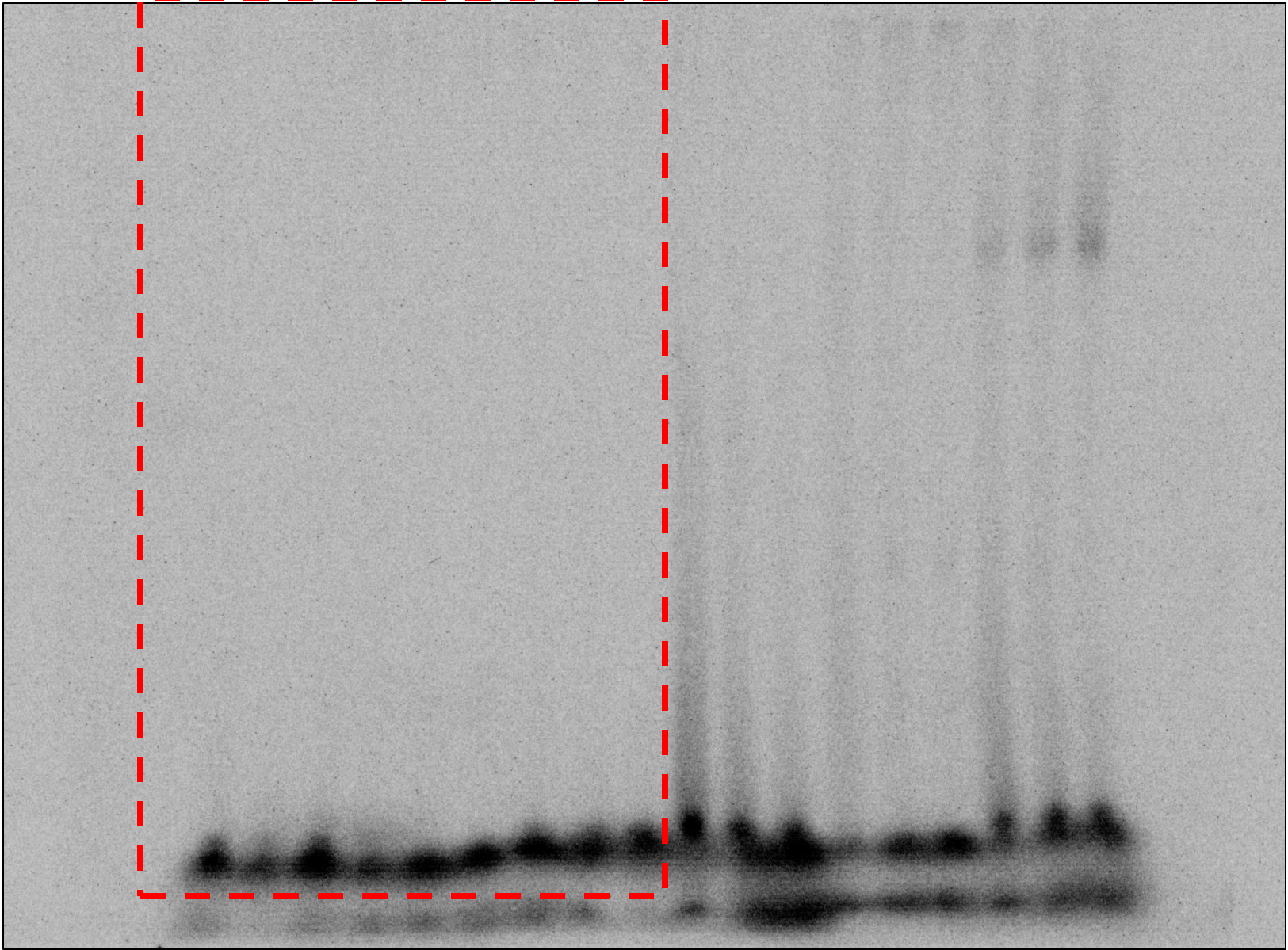

**Gel ii**

**Gel iv**

**ED Figure 4c**

Section presented

Supplement: Supplementary file 19 — Unprocessed gel image data for Extended Data Fig. 4. [file 41594_2025_1671_MOESM19_ESM.pdf]

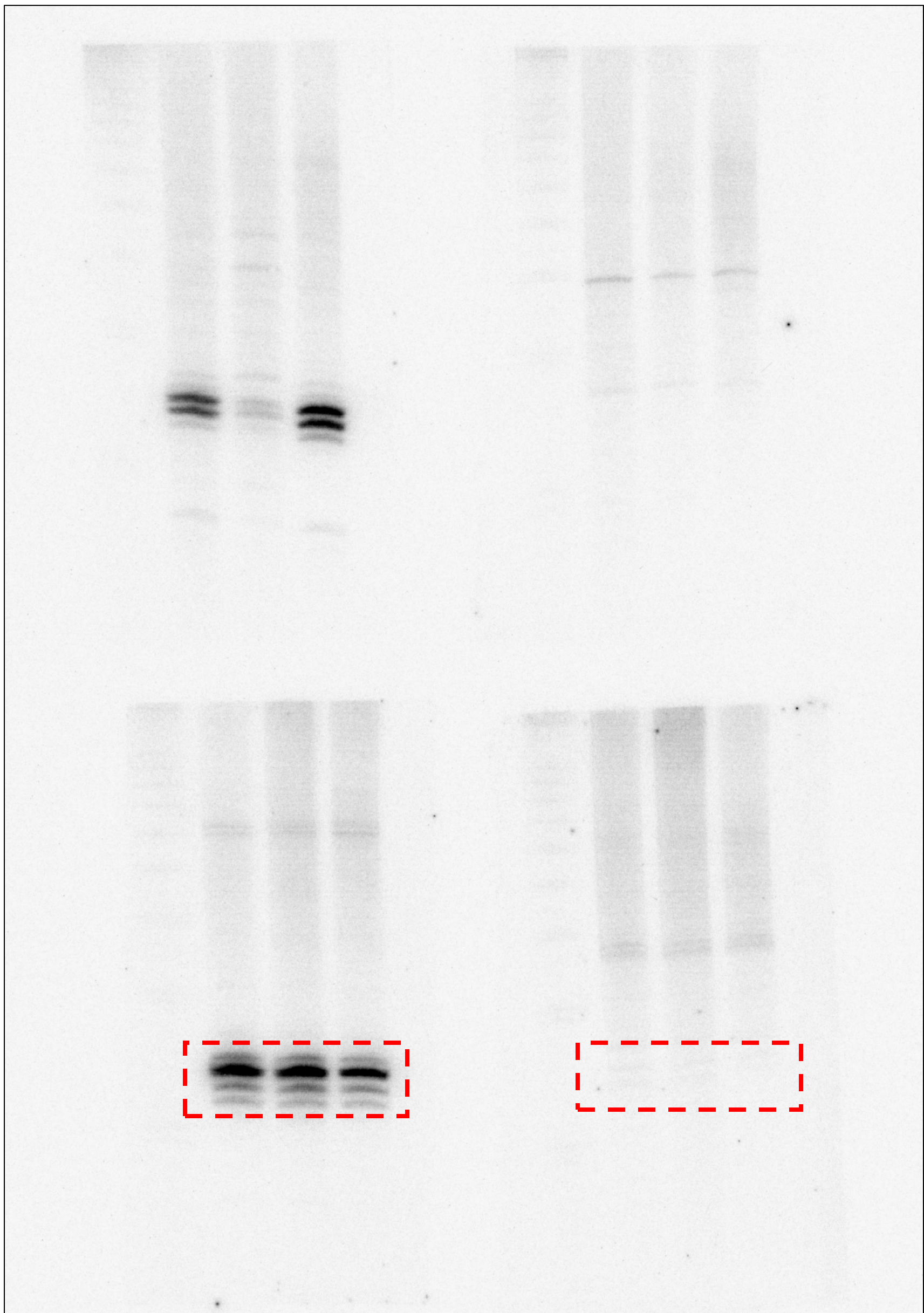

**ED Figure 5i (top panels)**

Section presented

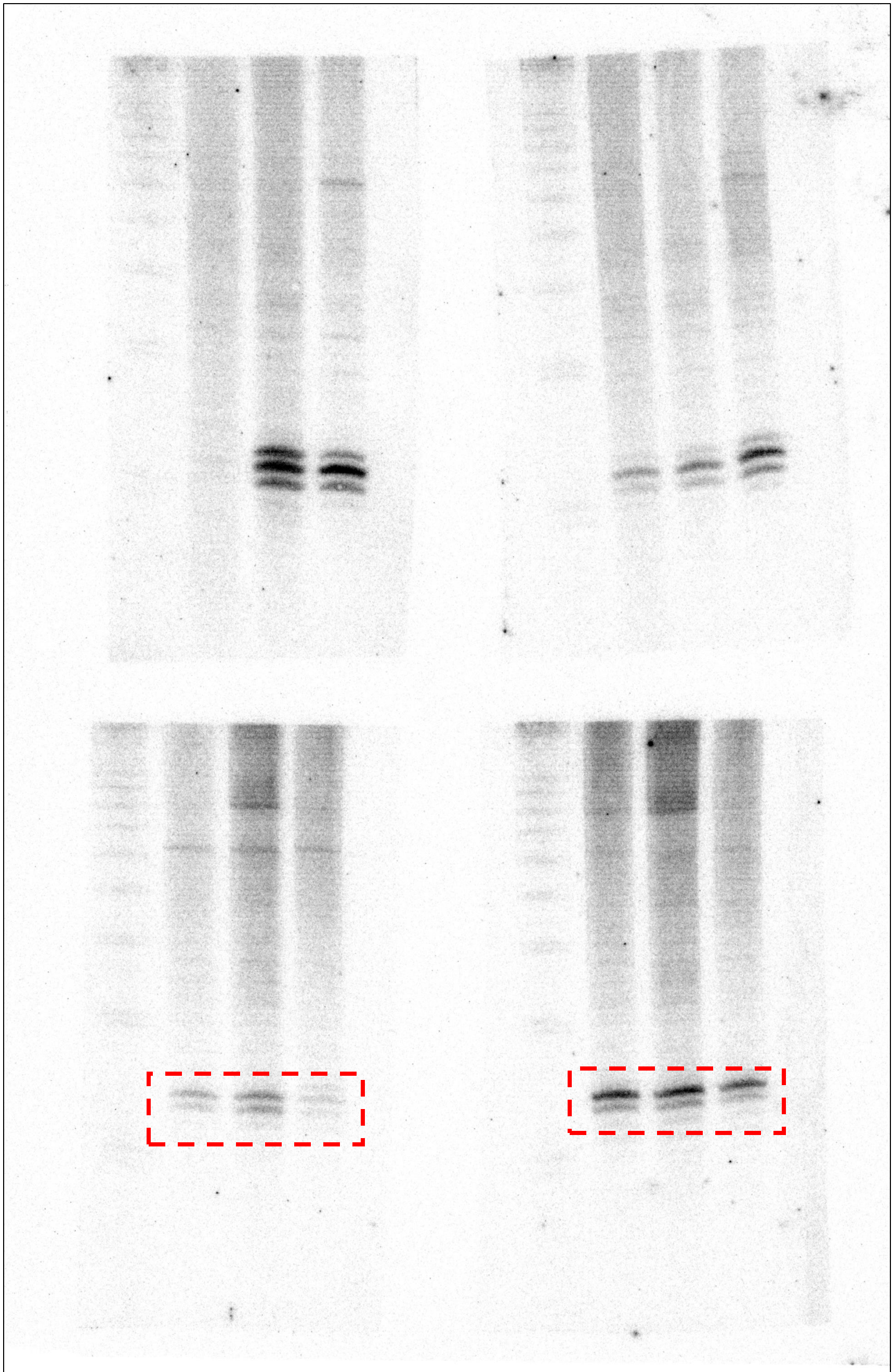

**ED Figure 5i (bottom panels)**

Section presented

Supplement: Supplementary file 21 — Unprocessed northern blot image data for Extended Data Fig. 5. [file 41594_2025_1671_MOESM21_ESM.pdf]
