# Supplementary material for: DICER1 hotspot mutation induces 3p microRNA gain of function via Argonaute strand switch
Source: Nat Struct Mol Biol. 2025 Nov 4;32(12):2542–52. doi: 10.1038/s41594-025-01671-w (PMC12700799; doi:10.1038/s41594-025-01671-w)
Supplement: Supplementary file 10 — Unprocessed northern blot and gel image data for Fig. 4. [file 41594_2025_1671_MOESM10_ESM.pdf]

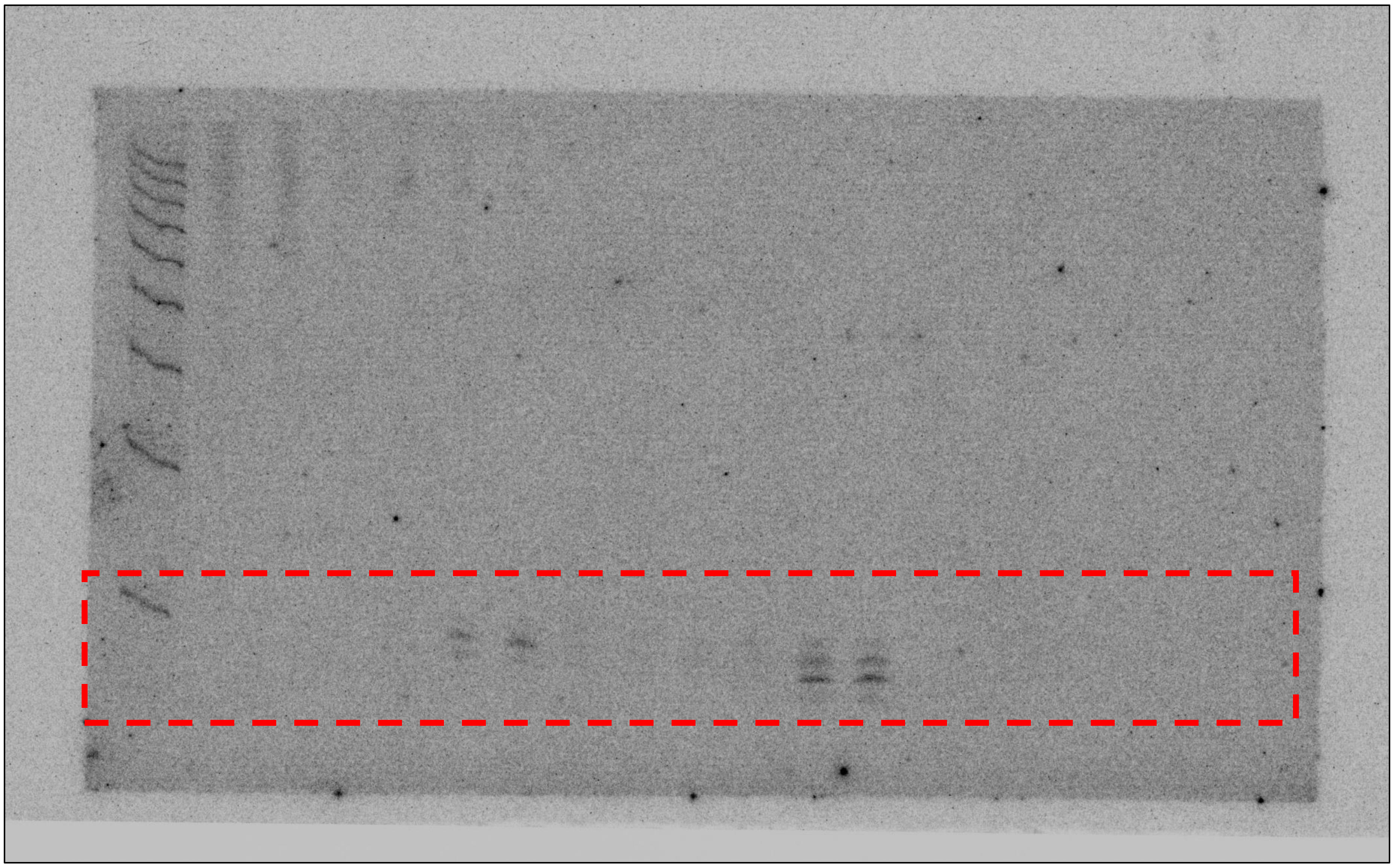

miR-7a-1-3p

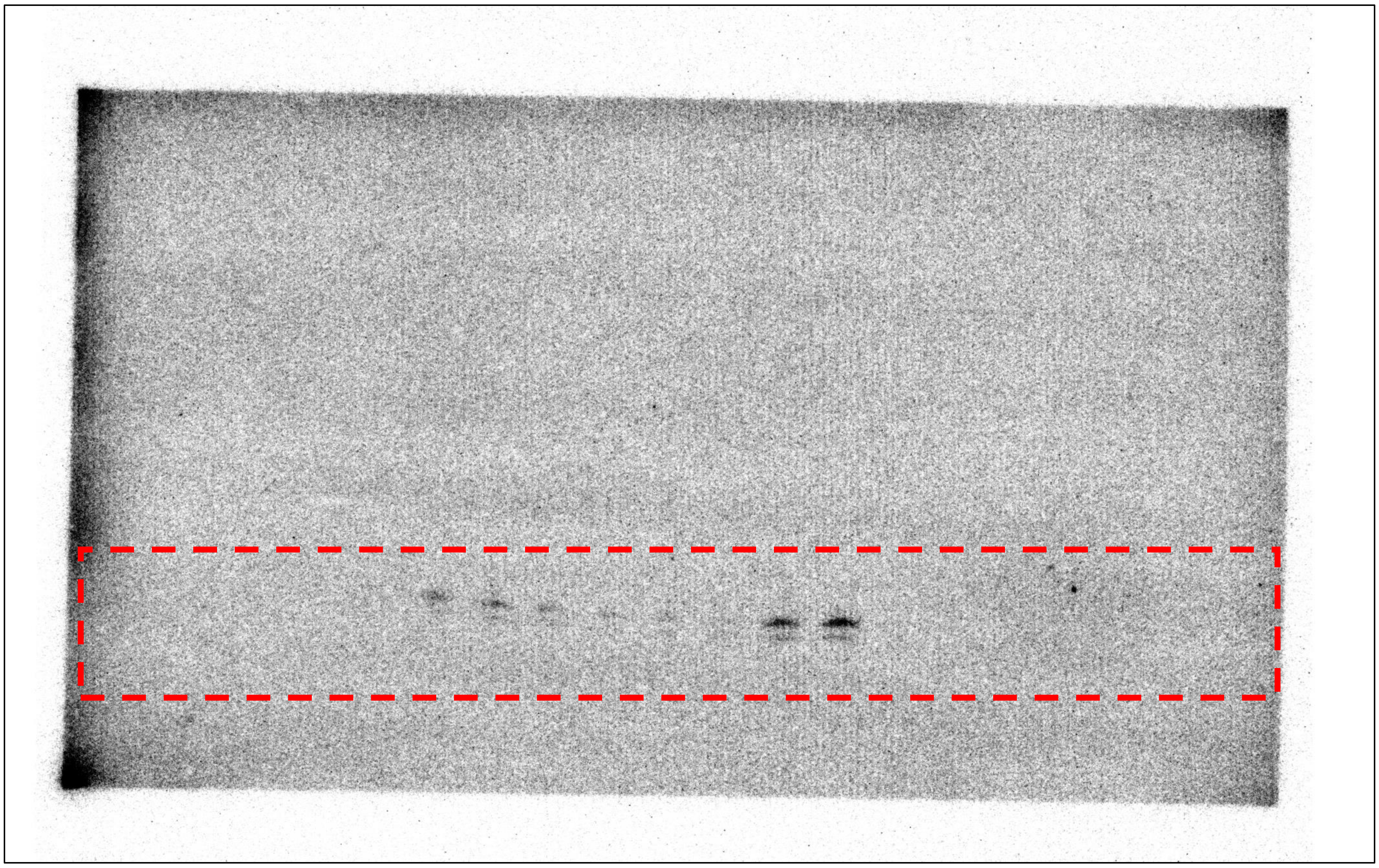

miR-17-3p

Figure 4a

Section presented

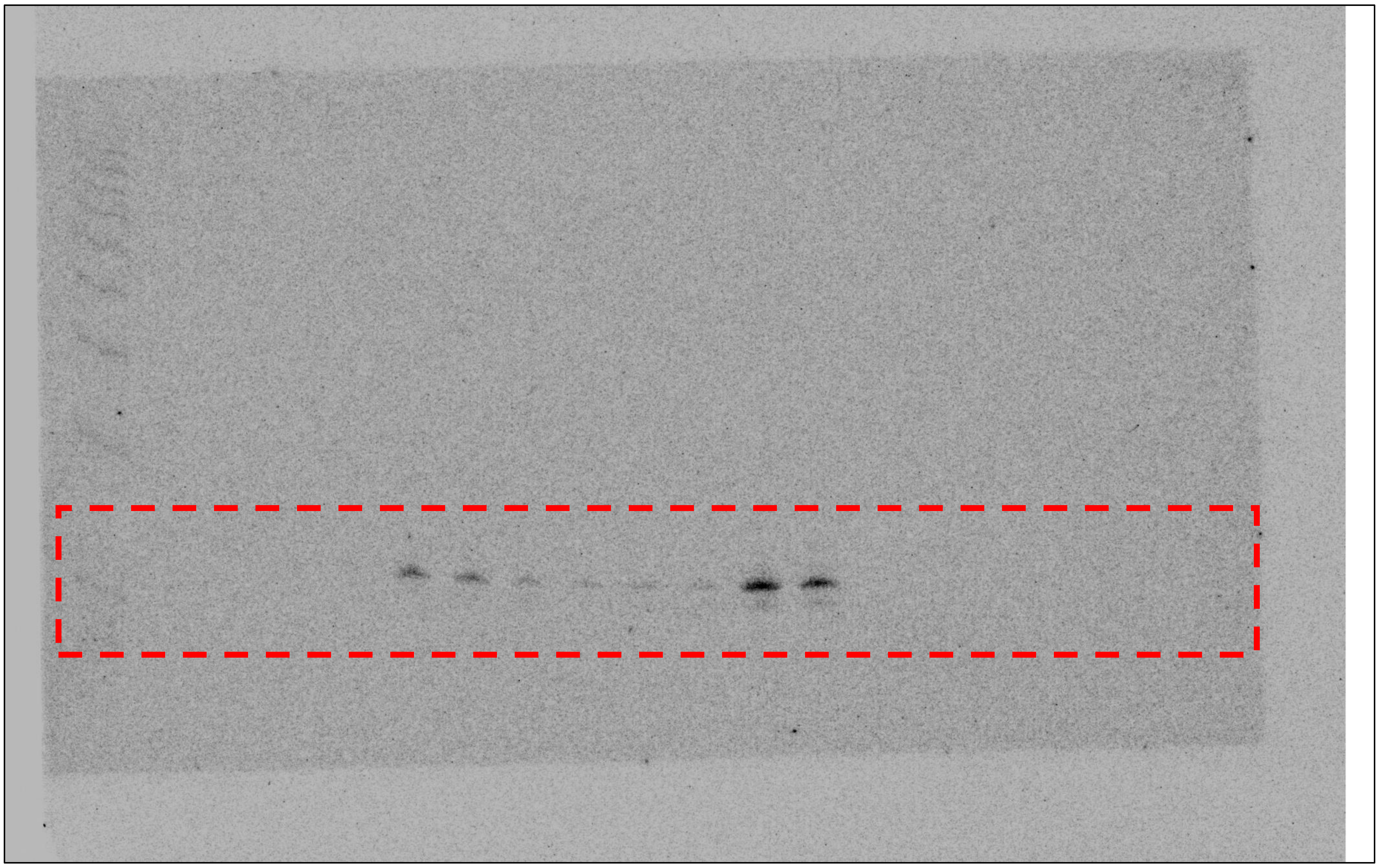

miR-20b-3p

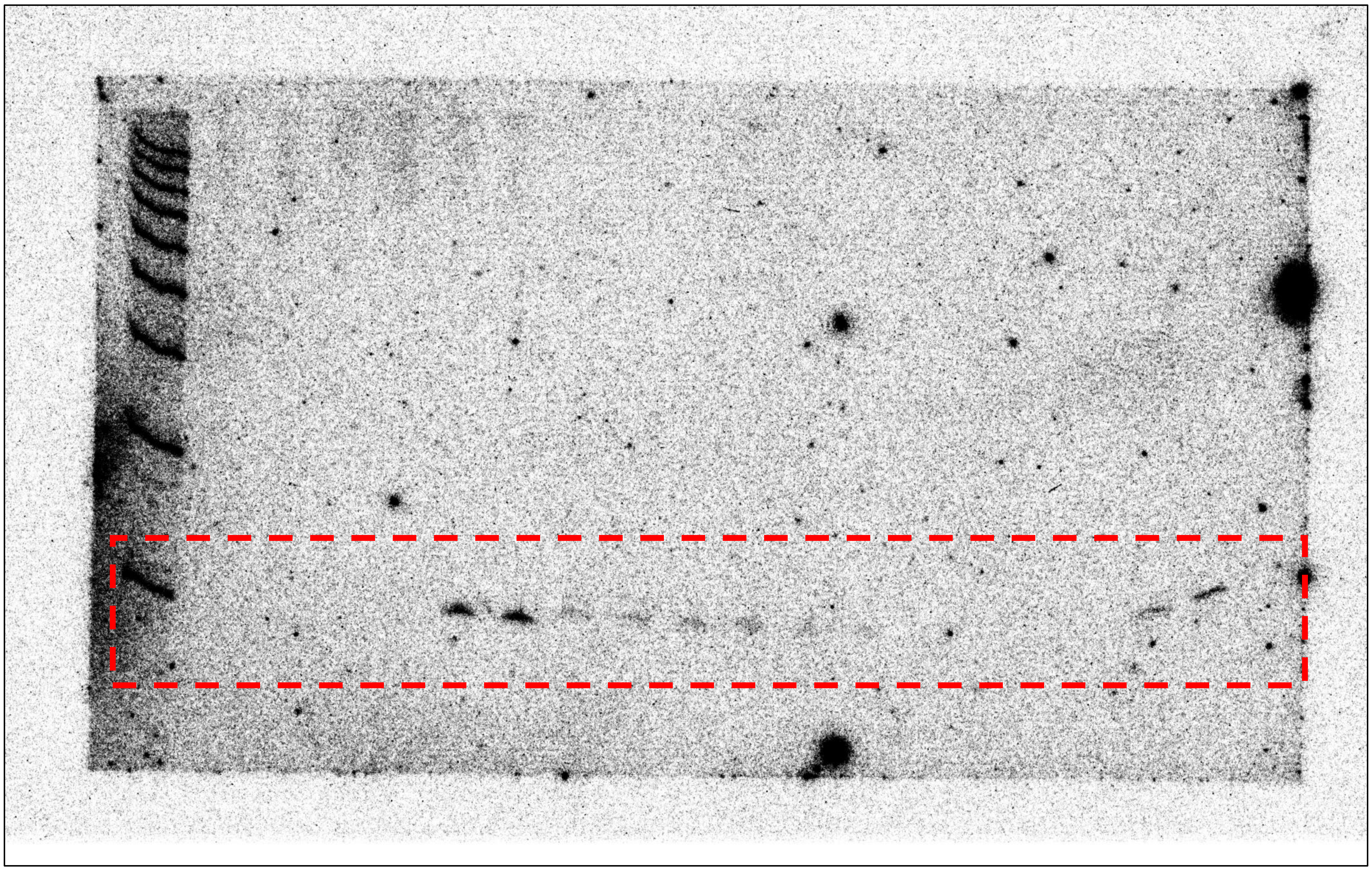

miR-26a-2-3p

Figure 4a (continued)

Section presented

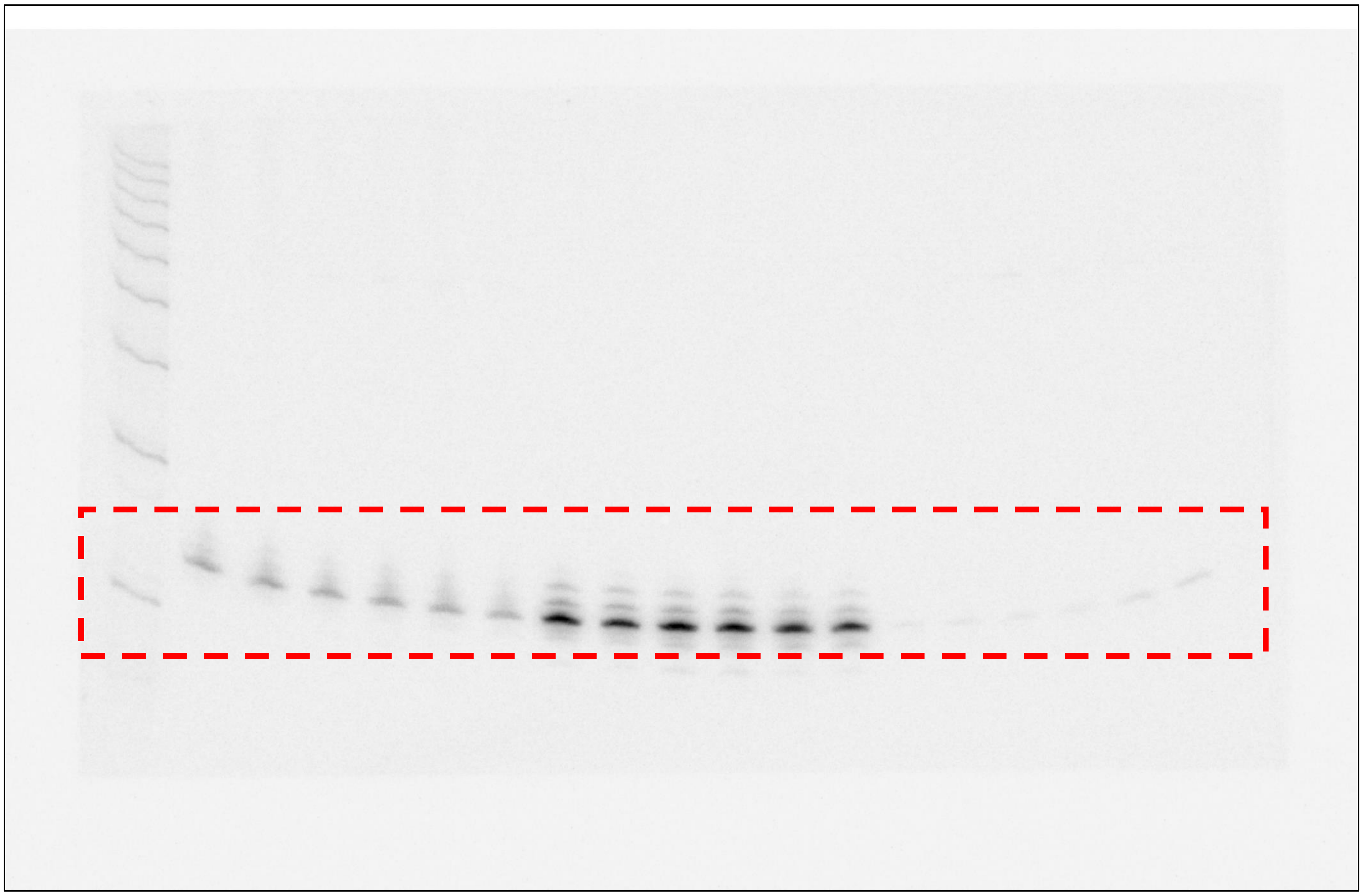

miR-92a-3p

Figure 4a (continued)

Section presented

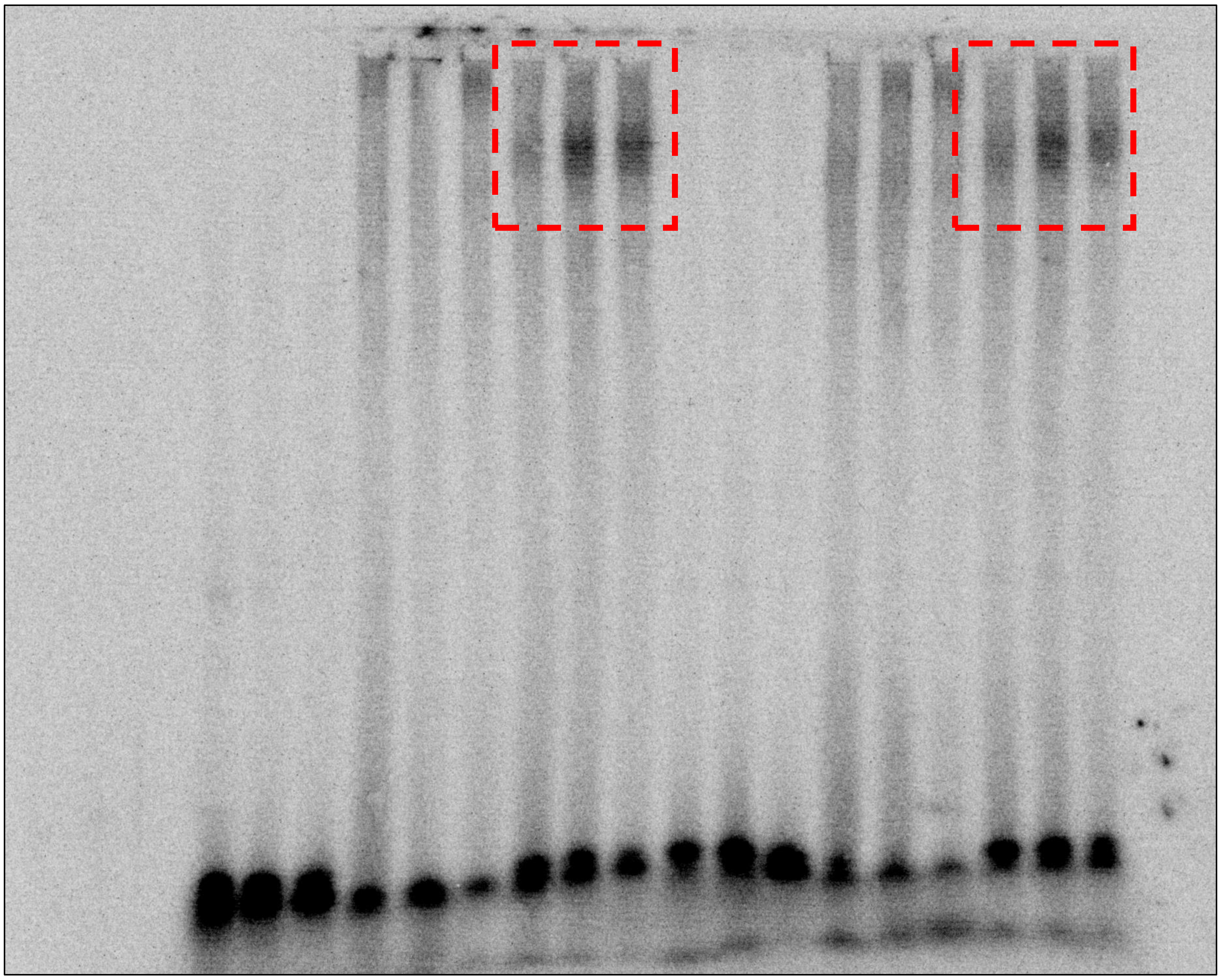

Gel i Gel iii

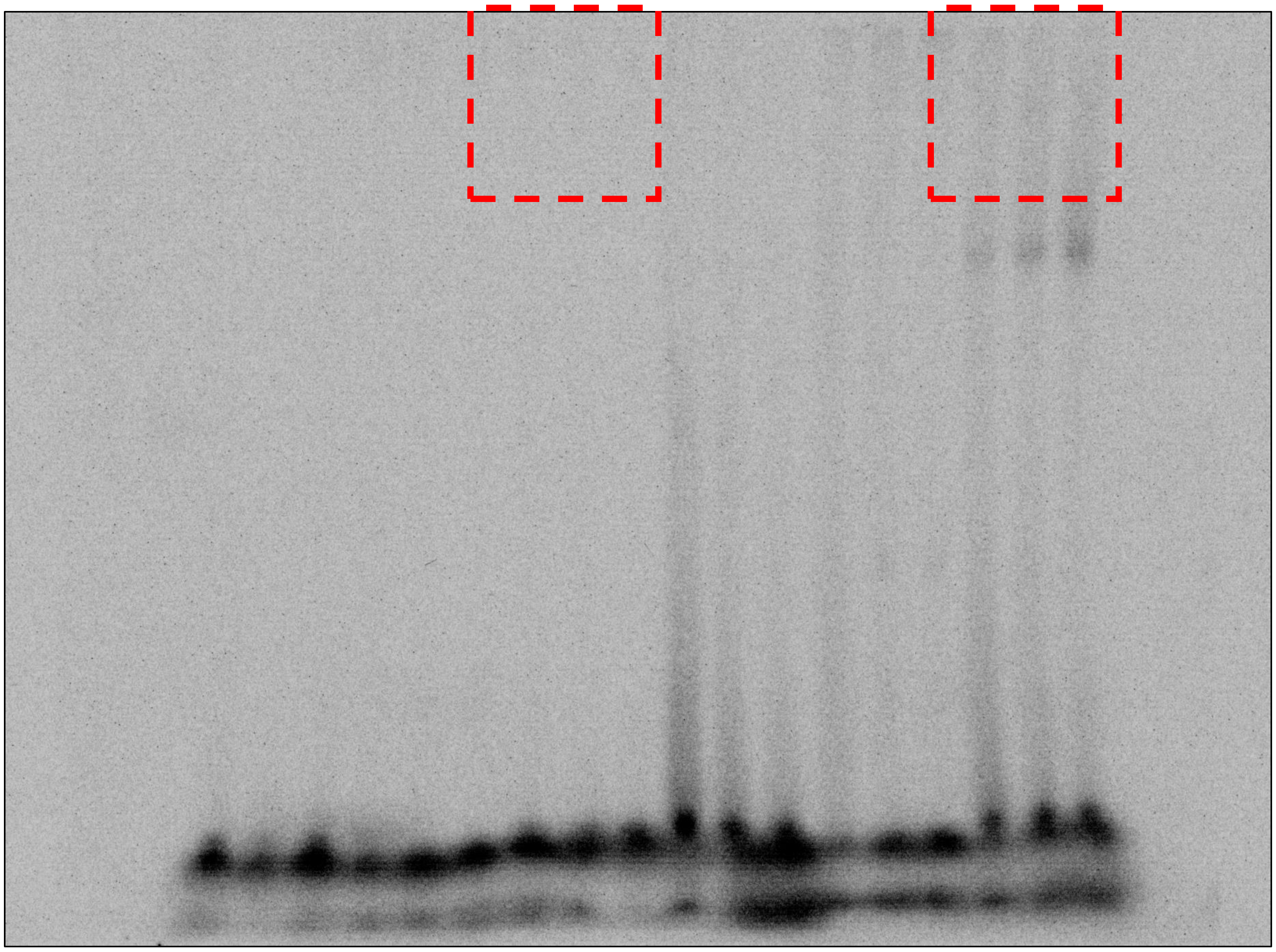

Gel ii Gel iv

Figure 4c

Section presented

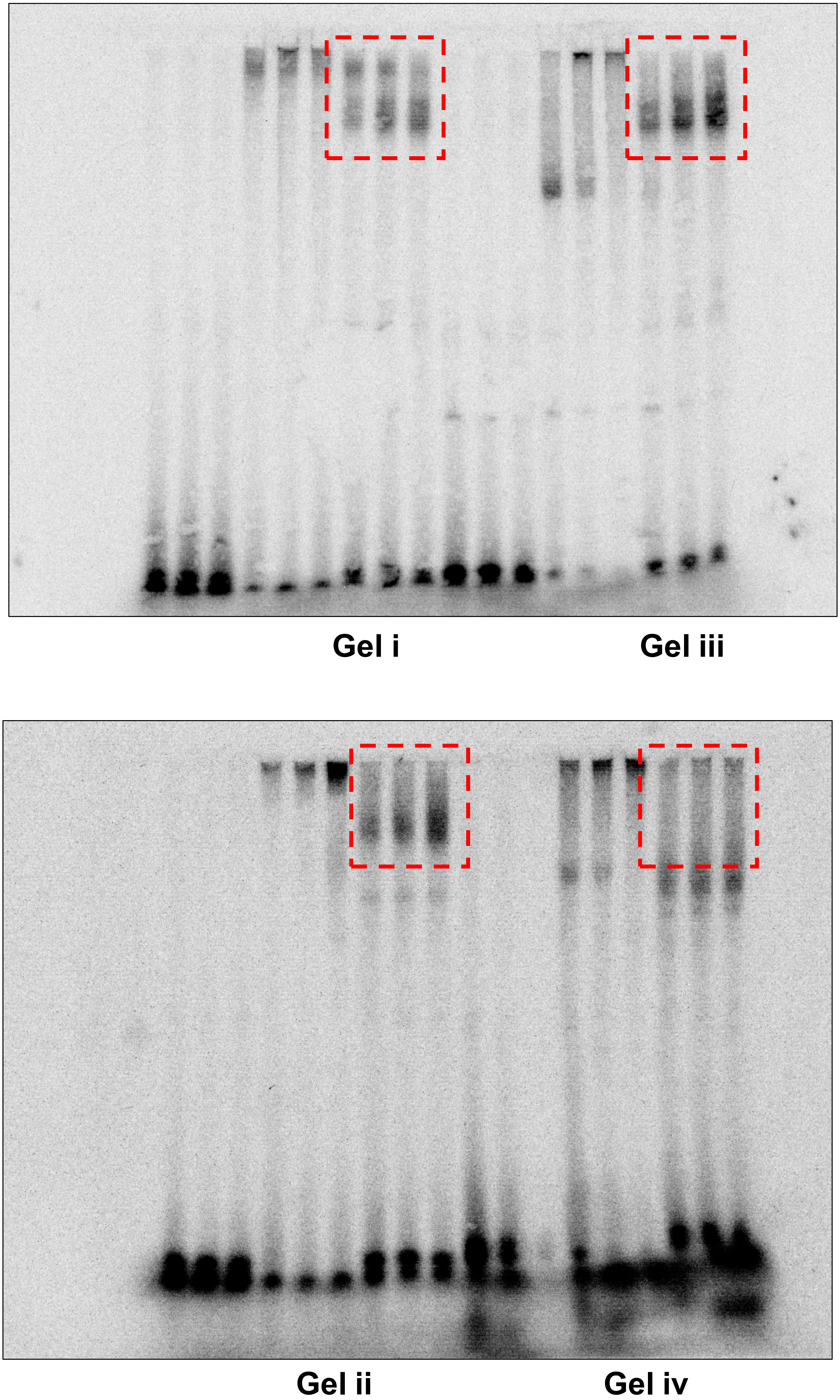

Figure 4d

Section presented

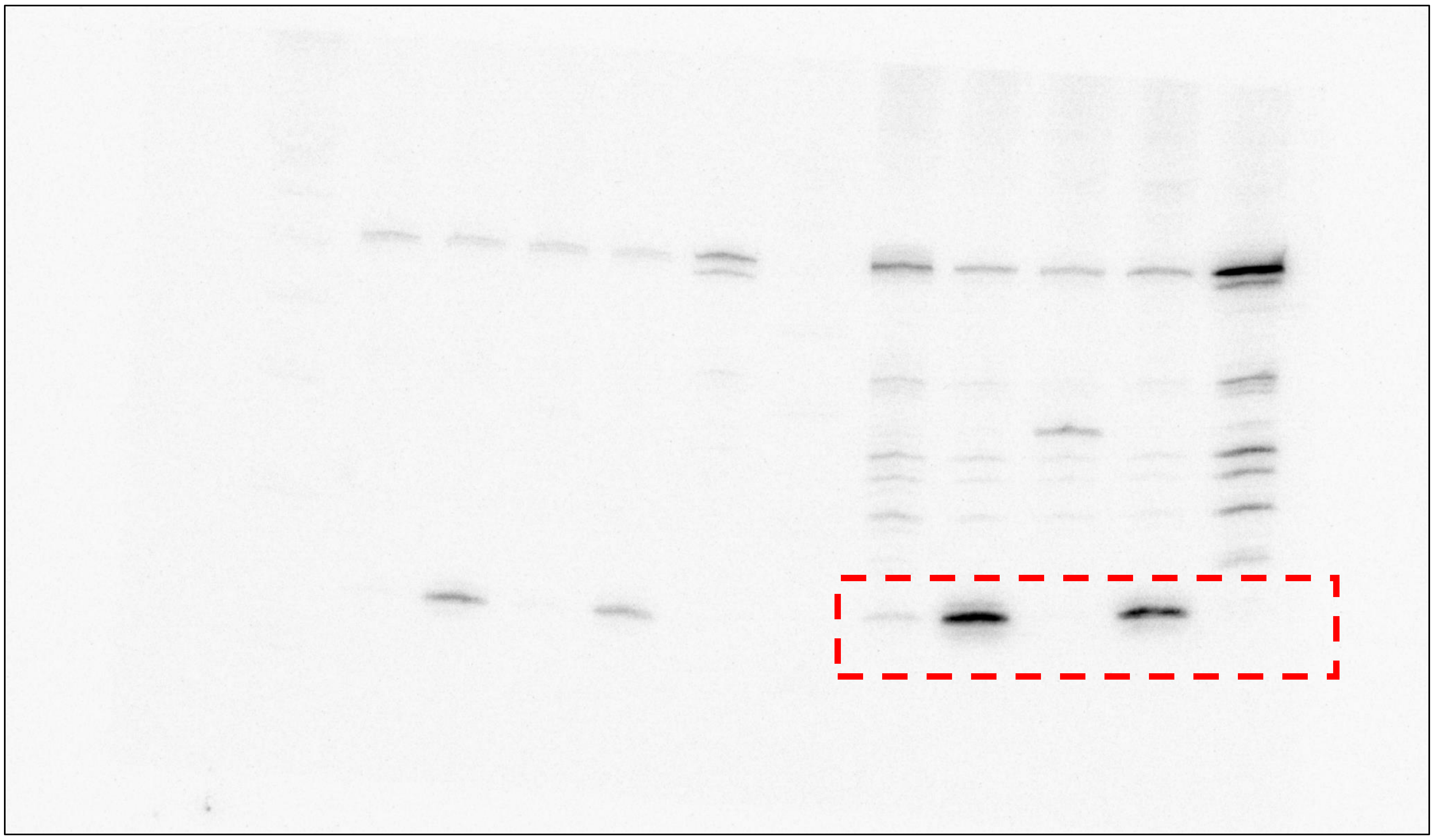

miR-19b-3p

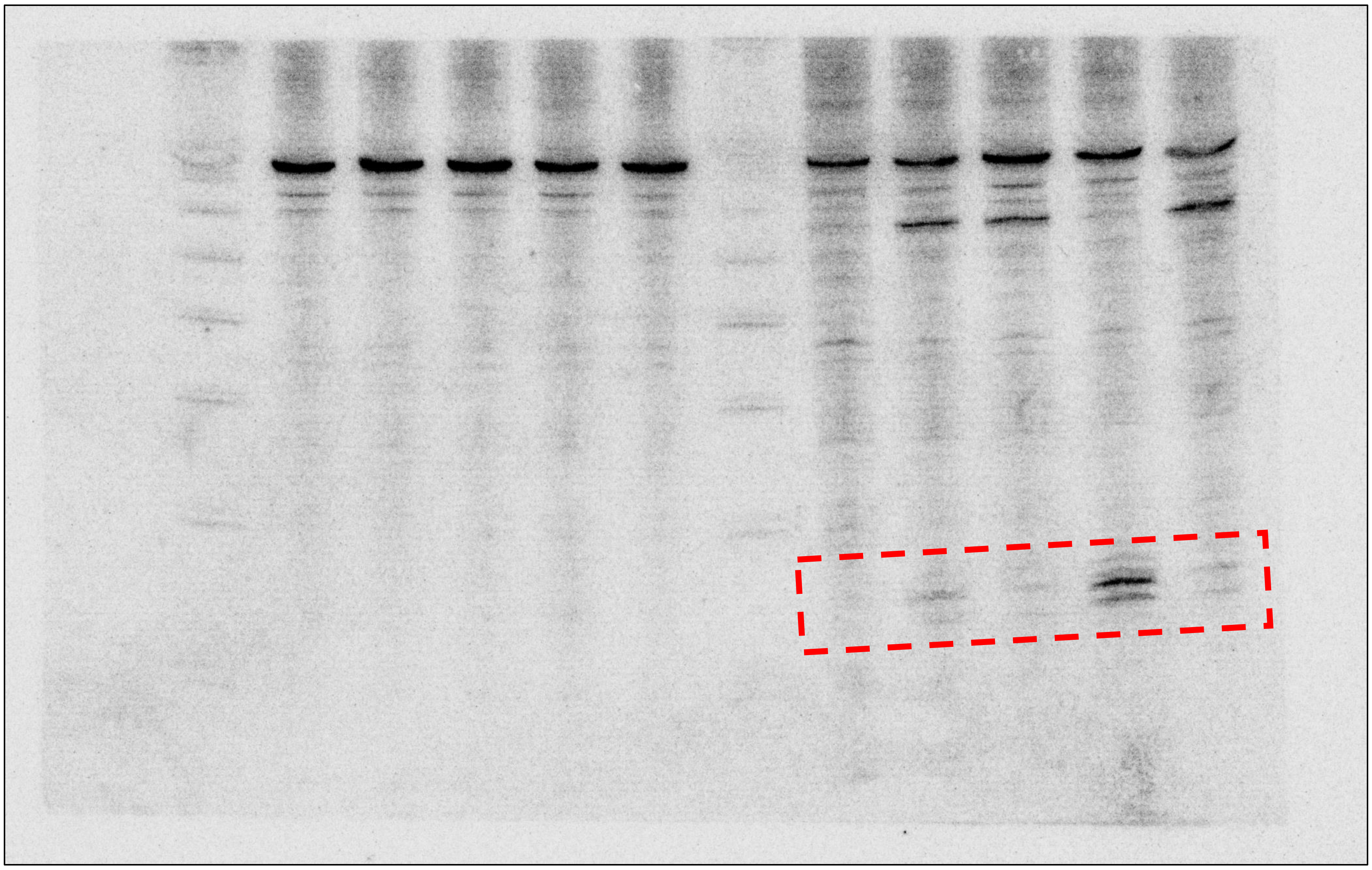

miR-99b-3p

Figure 4f

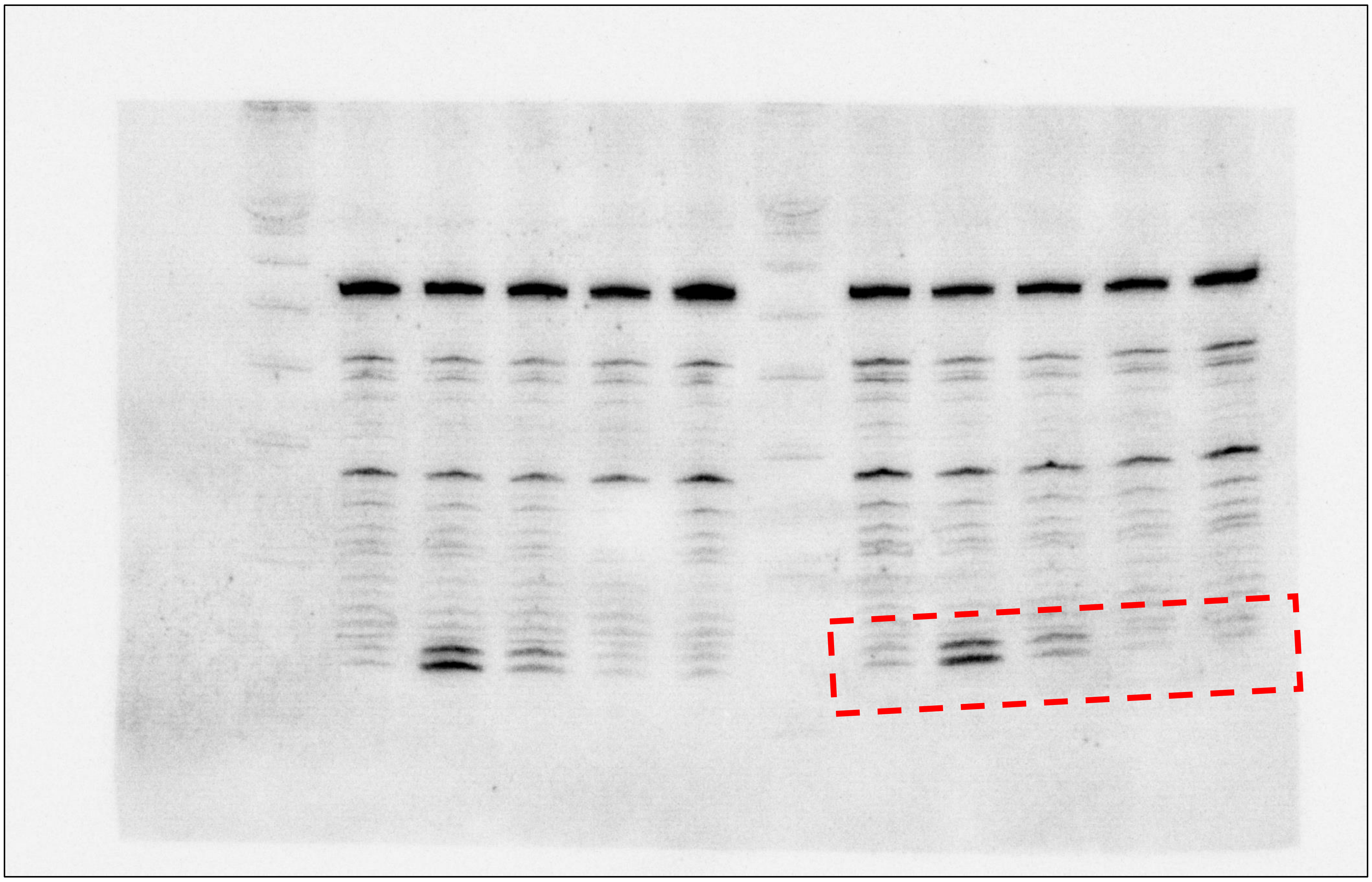

miR-16-5p

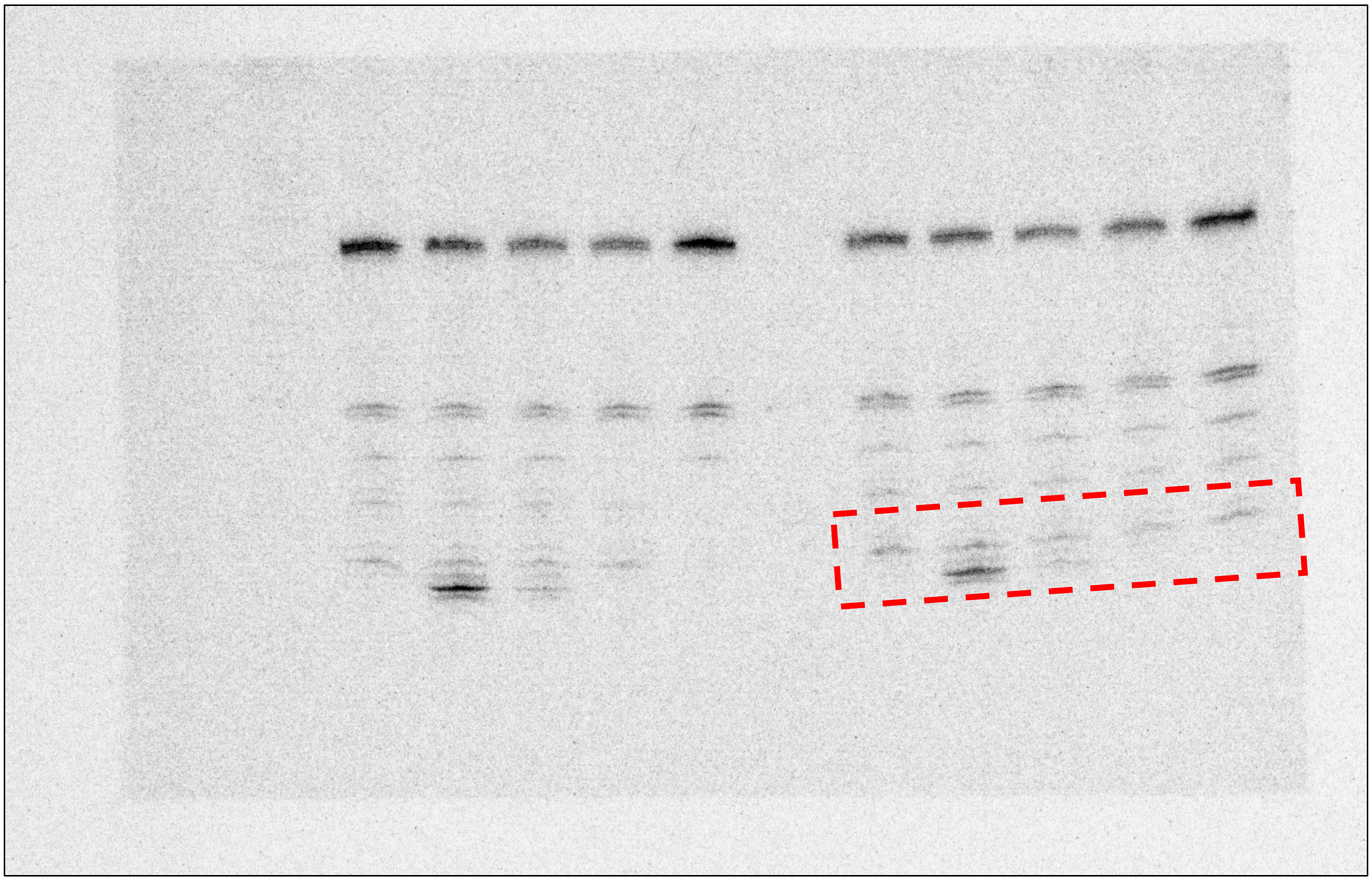

miR-7-5p

Figure 4f (continued)

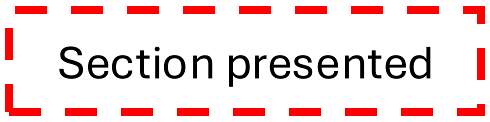

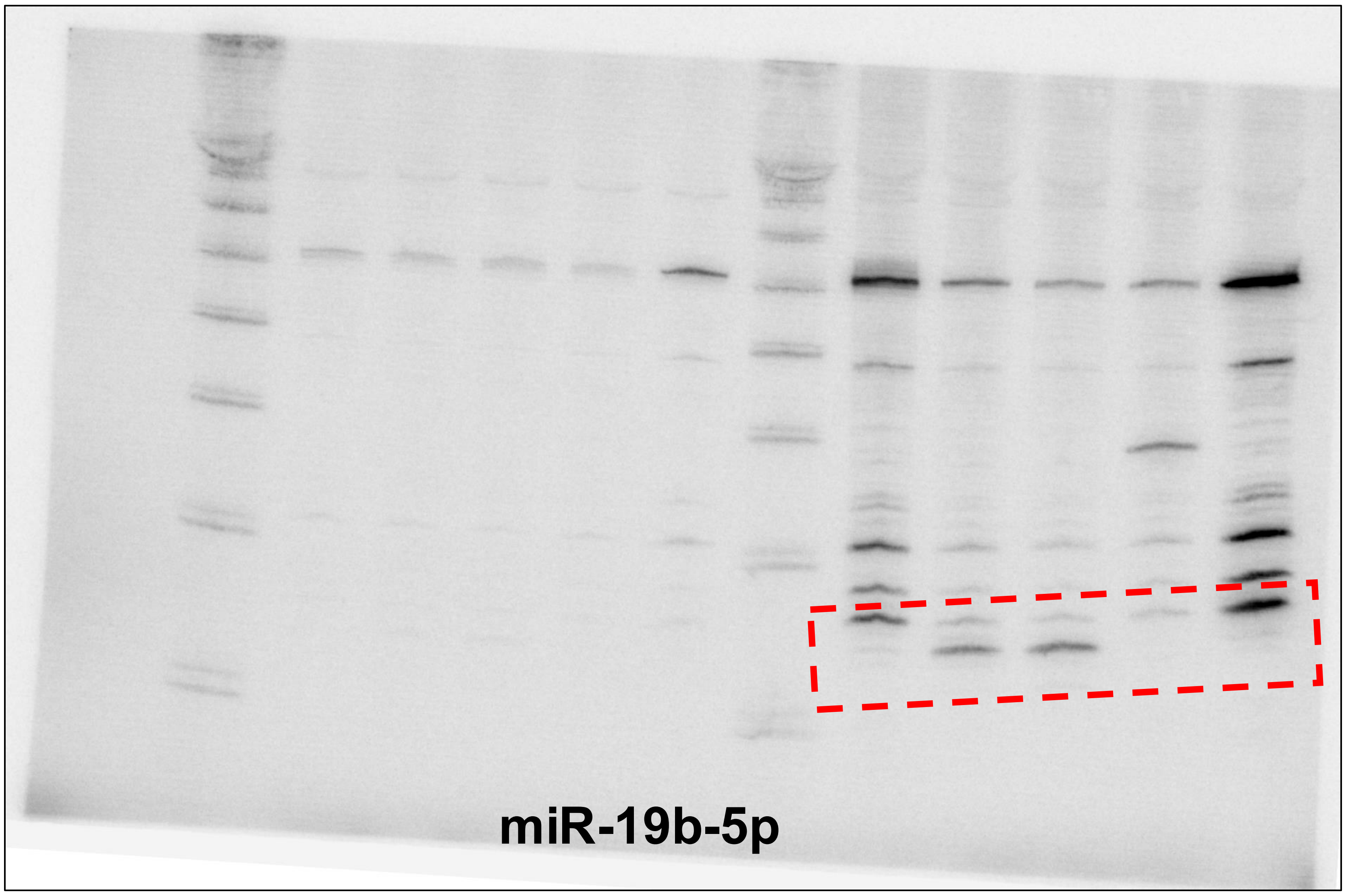

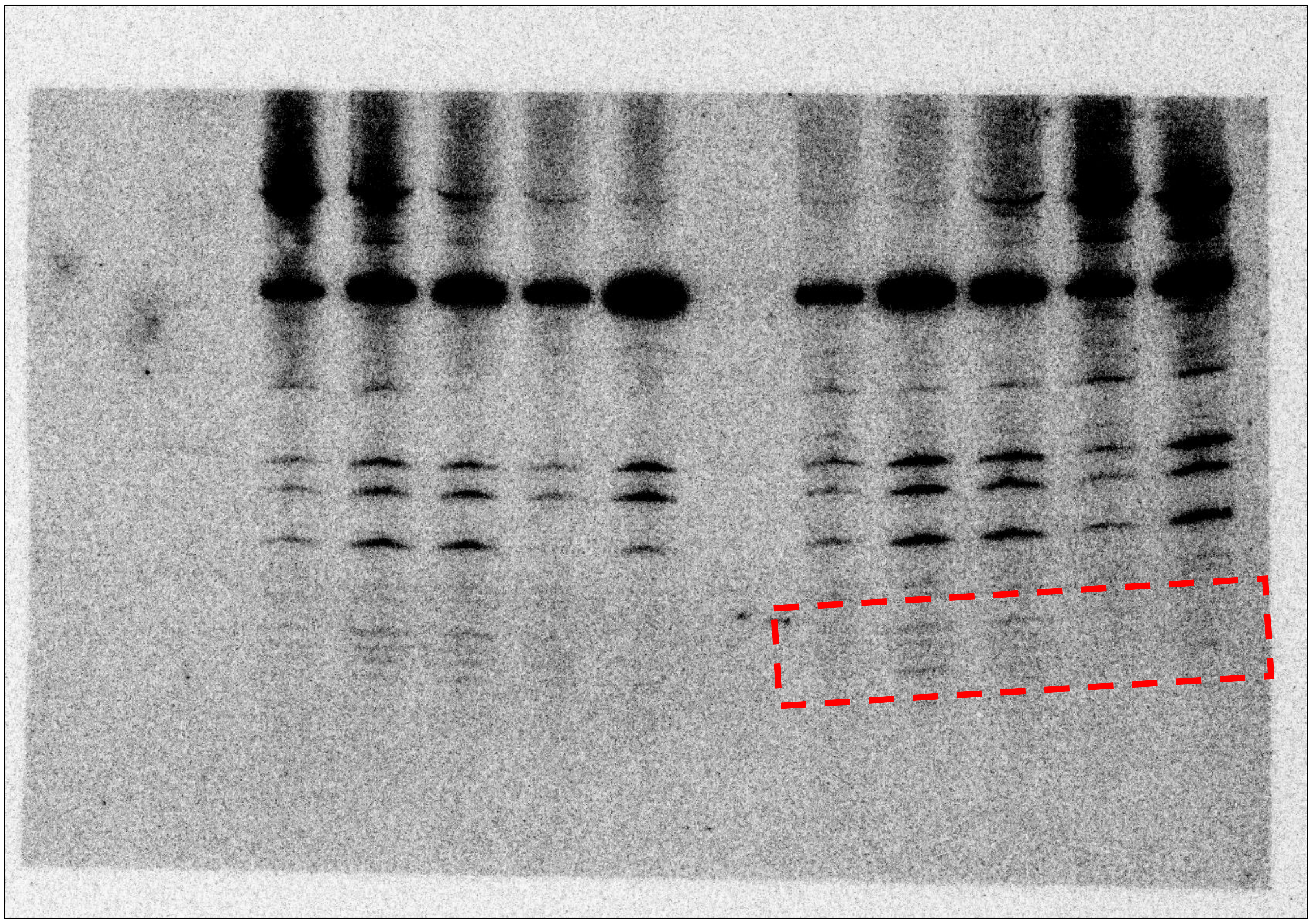

miR-221-5p

Figure 4f (continued)

Section presented
