# Supplementary material for: DICER1 hotspot mutation induces 3p microRNA gain of function via Argonaute strand switch
Source: Nat Struct Mol Biol. 2025 Nov 4;32(12):2542–52. doi: 10.1038/s41594-025-01671-w (PMC12700799; doi:10.1038/s41594-025-01671-w)
Supplement: Supplementary file 15 — Unprocessed northern blot, northern blot and gel image data for Extended Data Fig. 1. [file 41594_2025_1671_MOESM15_ESM.pdf]

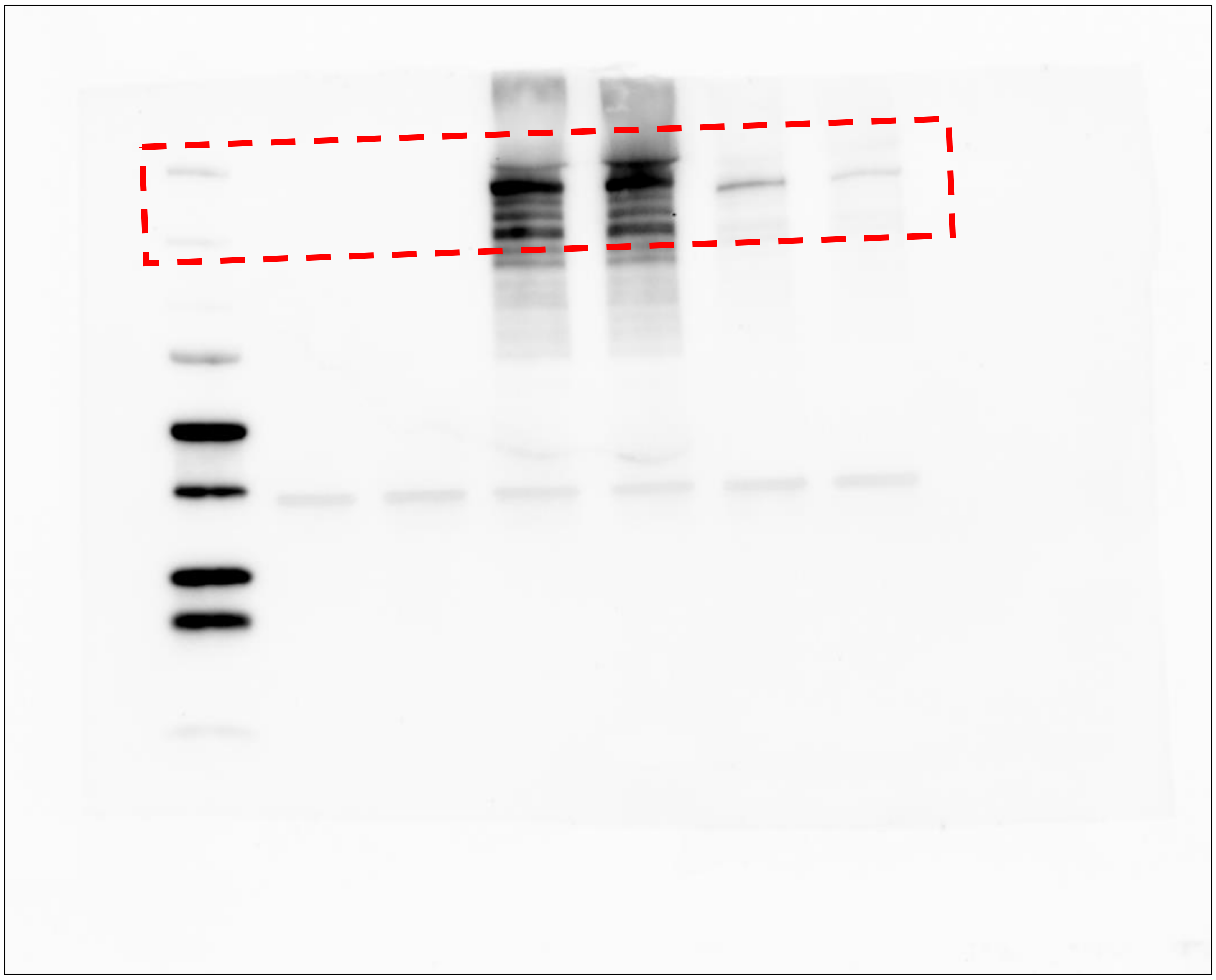

**Dicer**

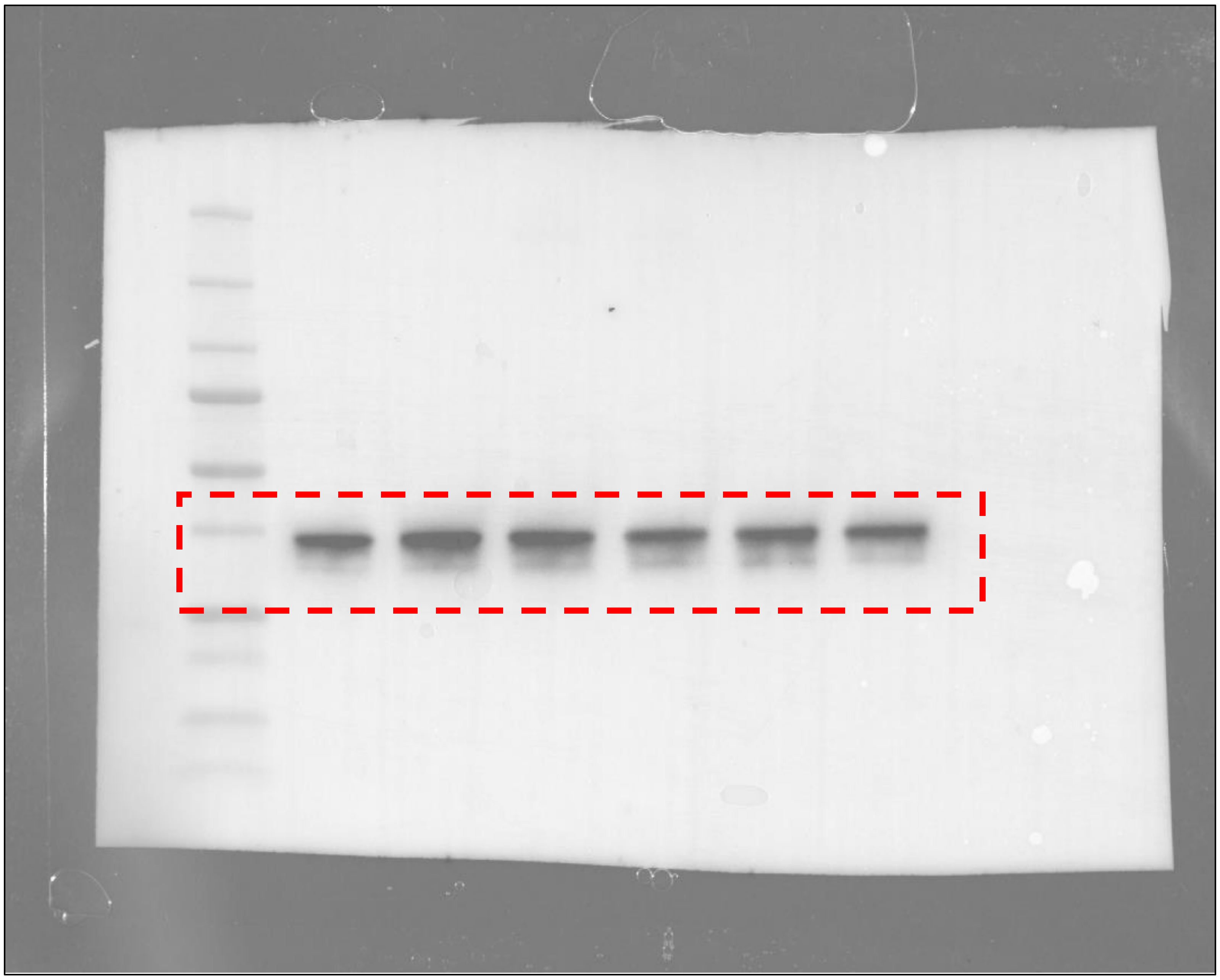

**α-tubulin**

# ED Figure 1b

Section presented

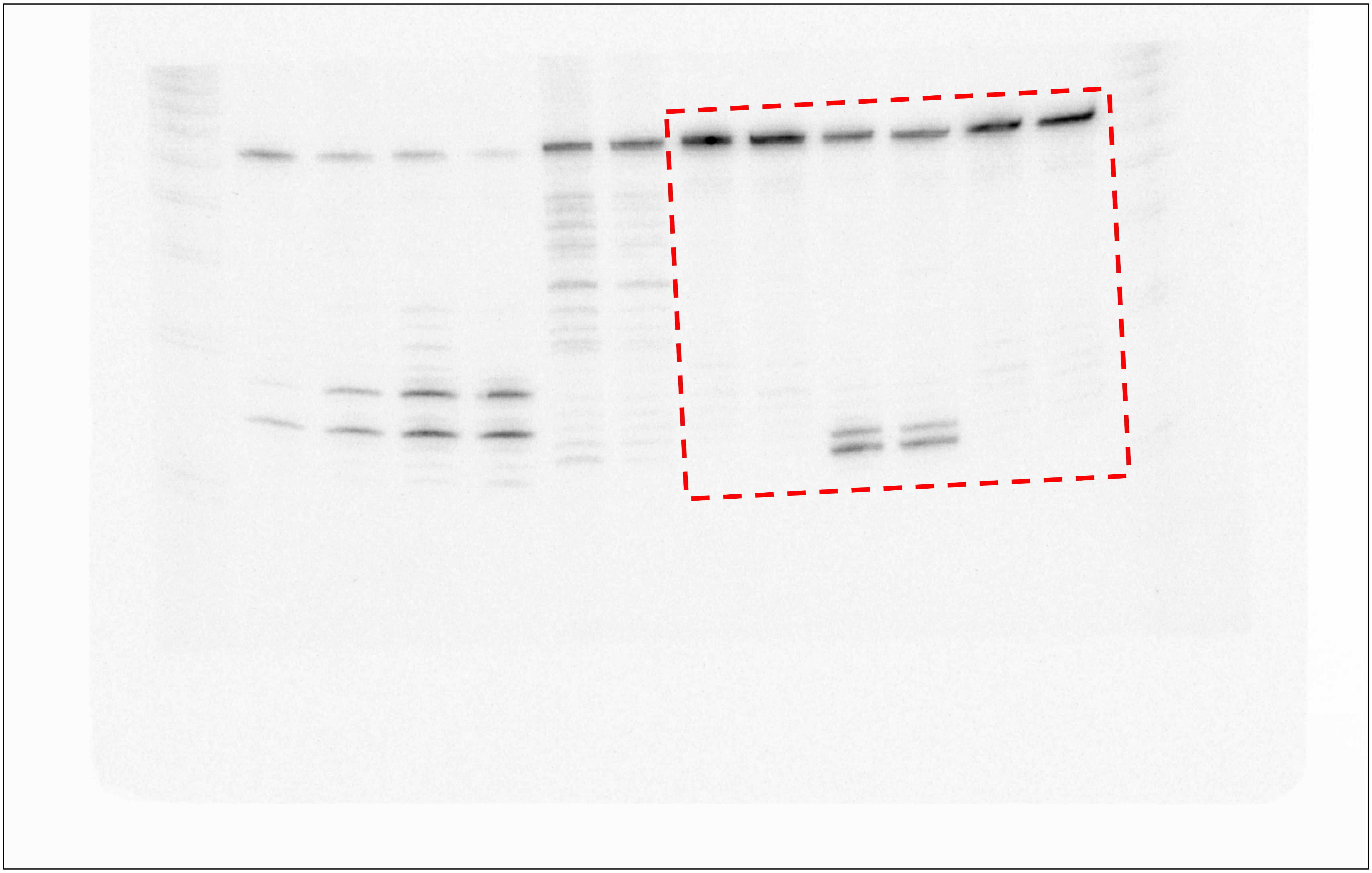

**miR-16-5p**

**ED Figure 1c**

Section presented

WT(+/+)

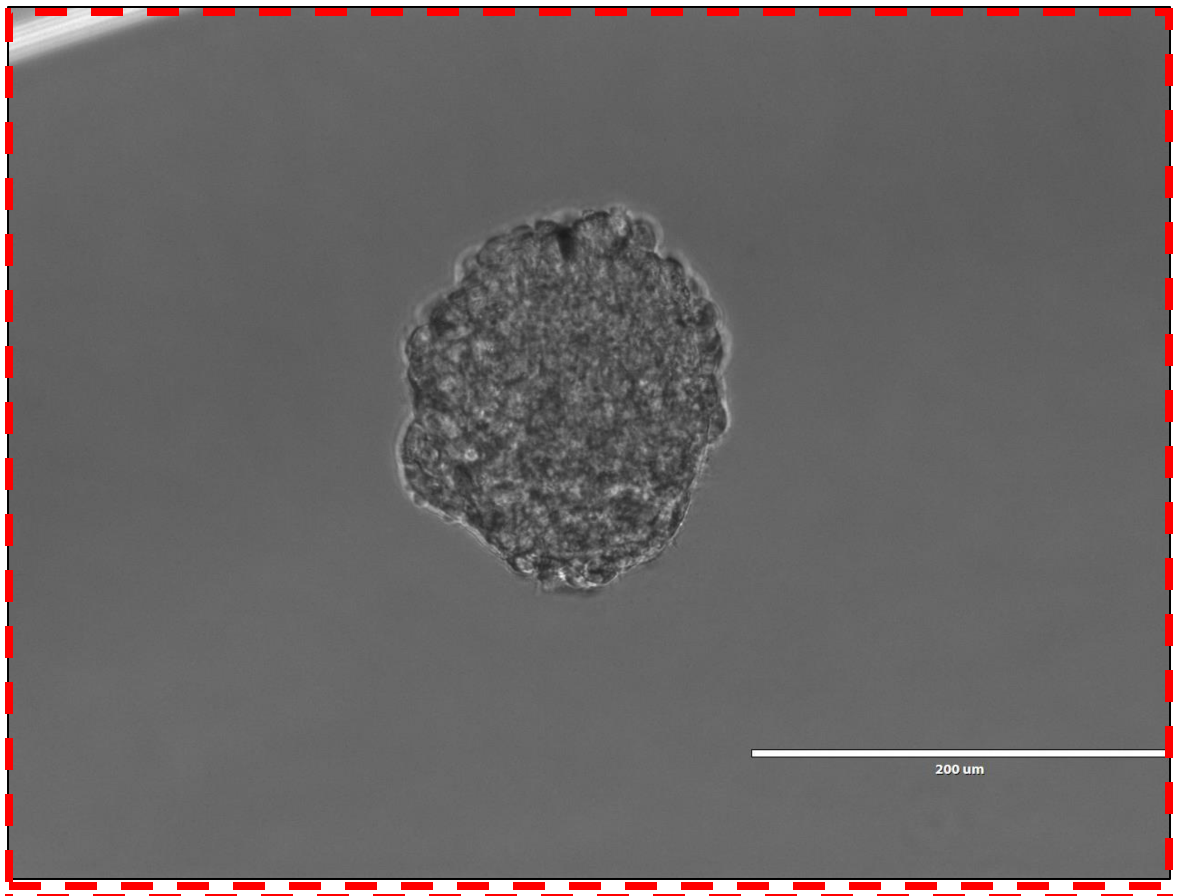


HetKO (+/-)

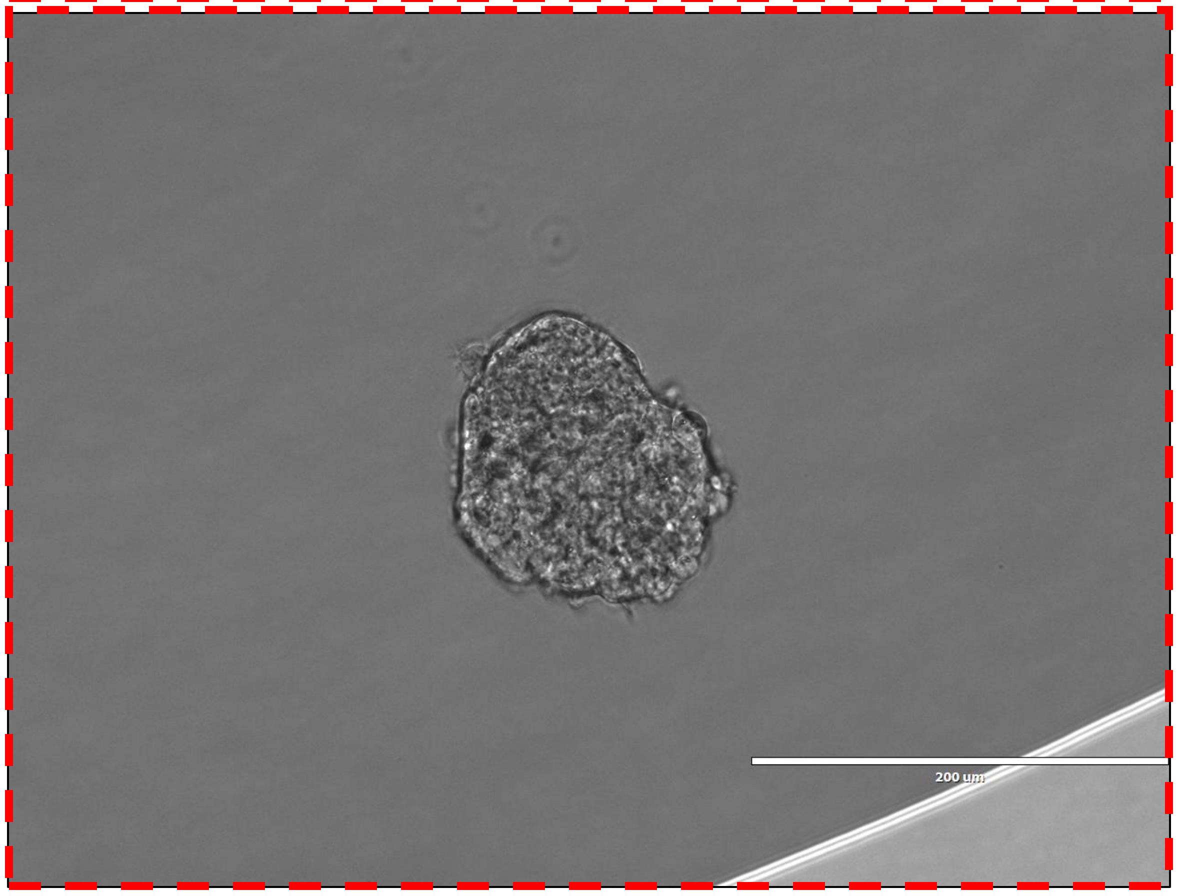


HM (HM/-)

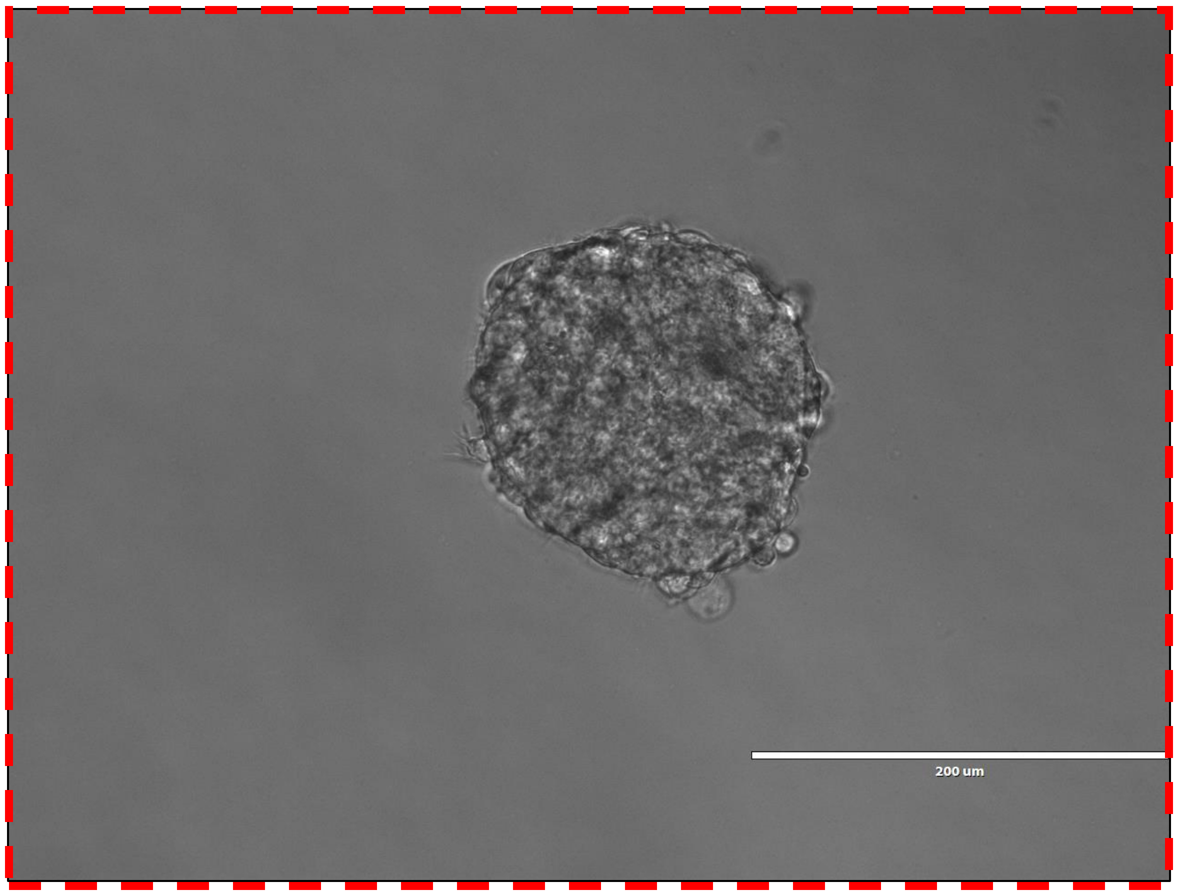


Day 2 spheroid

**ED Figure 1g (*entire image used*)**

Section presented

WT(+/+)

HetKO (+/-)

HM (HM/-)

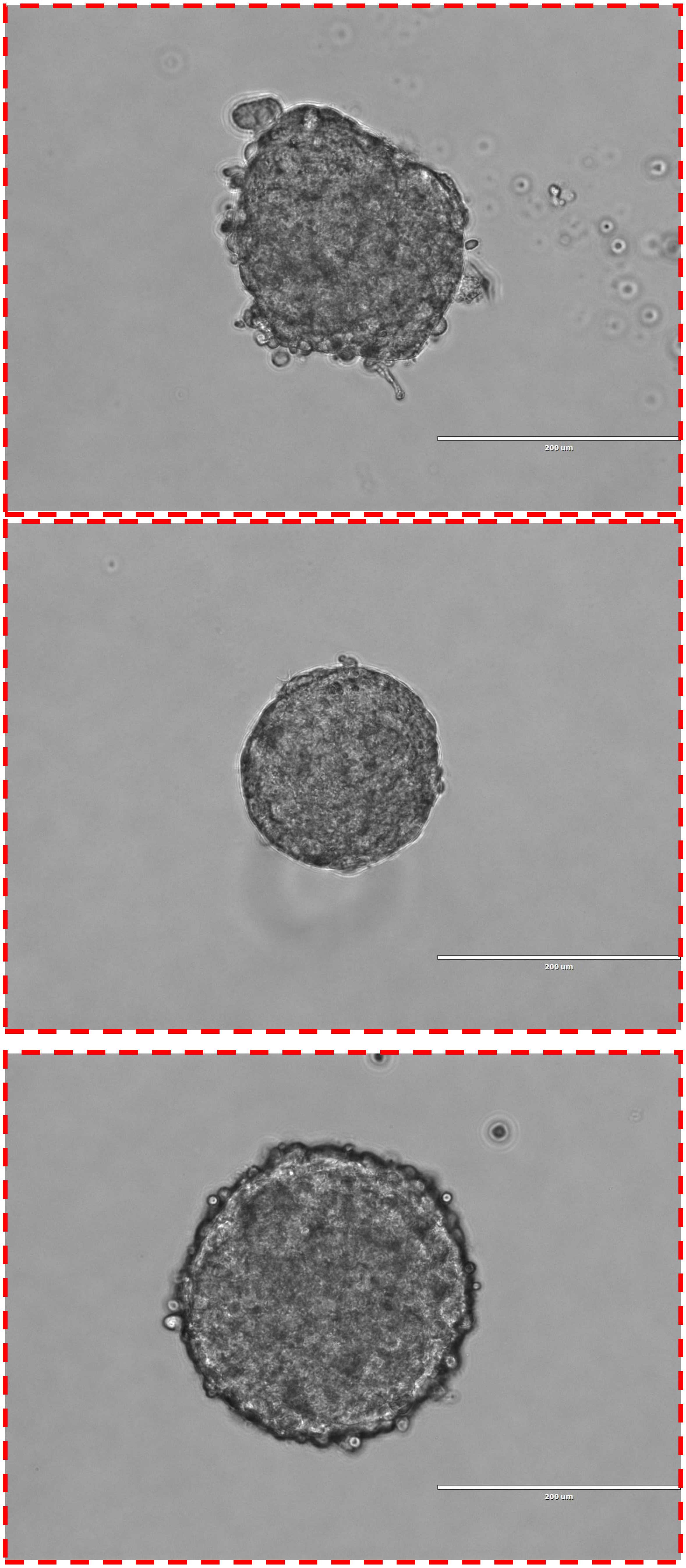


Day 3 spheroid

**ED Figure 1g (*entire image used*)**

Section presented
